# Prospective multicenter randomized patient recruitment and sample collection to enable future measurements of sputum biomarkers of inflammation in an observational study of cystic fibrosis

**DOI:** 10.1186/s12874-019-0705-0

**Published:** 2019-04-26

**Authors:** Theodore G. Liou, Frederick R. Adler, Natalia Argel, Fadi Asfour, Perry S. Brown, Barbara A. Chatfield, Cori L. Daines, Dixie Durham, Jessica A. Francis, Barbara Glover, Theresa Heynekamp, John R. Hoidal, Judy L. Jensen, Ruth Keogh, Carol M. Kopecky, Noah Lechtzin, Yanping Li, Jerimiah Lysinger, Osmara Molina, Craig Nakamura, Kristyn A. Packer, Katie R. Poch, Alexandra L. Quittner, Peggy Radford, Abby J. Redway, Scott D. Sagel, Shawna Sprandel, Jennifer L. Taylor-Cousar, Jane B. Vroom, Ryan Yoshikawa, John P. Clancy, J. Stuart Elborn, Kenneth N. Olivier, David R. Cox

**Affiliations:** 1 Adult Cystic Fibrosis Center, Division of Respiratory, Critical Care and Occupational Pulmonary Medicine, Department of Internal Medicine, University of Utah, 26 North Mario Capecchi Drive, Salt Lake City, UT 84132 USA; 2 Intermountain Pediatric Cystic Fibrosis Center, Division of Pediatric Pulmonology, Department of Pediatrics, University of Utah, 81 North Mario Capecchi Drive, Salt Lake City, UT 84113 USA; 3 Departments of Mathematics, University of Utah, 155 South 1400 east, JWB 233, Salt Lake City, UT 84112 USA; 4 School of Biological Sciences, University of Utah, 257 South 1400 East, Salt Lake City, UT 84112 USA; 5 Cystic Fibrosis Center, Phoenix Children’s Hospital, 1919 East Thomas Road, Phoenix, AZ 85016 USA; 6 St. Luke’s Cystic Fibrosis Center of Idaho, 610 W. Hays Street, Boise, ID 83702 USA; 7 Division of Pediatric Pulmonary and Sleep Medicine, Department of Pediatrics, University of Arizona Health Sciences, 1501 N. Campbell Avenue, Room 3301, PO Box 245073, Tucson, AZ 85724 USA; 8 Cystic Fibrosis Center, 3006 S. Maryland Pkwy, Suite #315, Las Vegas, NV 89109 USA; 9 Adult Cystic Fibrosis Program, Division of Pulmonary, Critical Care and Sleep Medicine, DoIM MSC10-5550, 1 University of New Mexico, Albuquerque, NM 87131 USA; 10 Department of Medical Statistics, London School of Hygiene and Tropical Medicine, Room G36, Keppel Street, London, WC1E 7HT UK; 11 Department of Pediatrics, Children’s Hospital Colorado, University of Colorado School of Medicine, 13123 East 16th Avenue, Aurora, CO 80045 USA; 12 Division of Pulmonary and Critical Care and Sleep Medicine, Department of Medicine, Johns Hopkins University School of Medicine, 601 N. Caroline St, Baltimore, MD 21287 USA; 13 Montana Cystic Fibrosis Center, Billings Clinic, 2800 10th Avenue N, Billings, MT 59101 USA; 14 Division of Pulmonary and Critical Care and Sleep Medicine, Department of Medicine, National Jewish Health, 1400 Jackson Street, Denver, CO 80206 USA; 15 Former: Department of Psychology, University of Miami, Miami, FL USA; 16 Present Address: Miami Children’s Research Institute, Nicklaus Children’s Hospital, 3100 SW 62nd Ave, Miami, FL 33155 USA; 17 Division of Pulmonology, Department of Pediatrics, National Jewish Health, 1400 Jackson St, Denver, CO 80206 USA; 18 Division of Pulmonary Medicine, Department of Pediatrics, University of Cincinnati, 3333 Burnet Avenue, Cincinnati, OH 45229-3026 USA; 19 Faculty of Medicine, Health and Life Sciences, Queen’s University Belfast, 90 Lisburn Road, Belfast, BT9 6AG UK; 20 Laboratory of Chronic Airway Infection, Pulmonary Branch, National Heart Lung and Blood Institute, National Institutes of Health, 10 Center Drive MSC1454, Building 10-CRC, Room 1408A, Bethesda, MD 20892 USA; 21 Nuffield College, 1 New Rd, Oxford, OX1 1NF UK

**Keywords:** Cystic fibrosis, Randomized observational trial, Study design, Sputum inflammation, HMGB-1, Neutrophil elastase, Calprotectin, Cystic Fibrosis Foundation patient registry

## Abstract

**Background:**

Biomarkers of inflammation predictive of cystic fibrosis (CF) disease outcomes would increase the power of clinical trials and contribute to better personalization of clinical assessments. A representative patient cohort would improve searching for believable, generalizable, reproducible and accurate biomarkers.

**Methods:**

We recruited patients from Mountain West CF Consortium (MWCFC) care centers for prospective observational study of sputum biomarkers of inflammation. After informed consent, centers enrolled randomly selected patients with CF who were clinically stable sputum producers, 12 years of age and older, without previous organ transplantation.

**Results:**

From December 8, 2014 through January 16, 2016, we enrolled 114 patients (53 male) with CF with continuing data collection. Baseline characteristics included mean age 27 years (SD = 12), 80% predicted forced expiratory volume in 1 s (SD = 23%), 1.0 prior year pulmonary exacerbations (SD = 1.2), home elevation 328 m (SD = 112) above sea level. Compared with other patients in the US CF Foundation Patient Registry (CFFPR) in 2014, MWCFC patients had similar distribution of sex, age, lung function, weight and rates of exacerbations, diabetes*,* pancreatic insufficiency, CF-related arthropathy and airway infections including methicillin-sensitive or -resistant *Staphylococcus aureus*, *Pseudomonas aeruginosa, Burkholderia cepacia* complex, fungal and non-tuberculous *Mycobacteria* infections. They received CF-specific treatments at similar frequencies.

**Conclusions:**

Randomly-selected, sputum-producing patients within the MWCFC represent sputum-producing patients in the CFFPR. They have similar characteristics, lung function and frequencies of pulmonary exacerbations, microbial infections and use of CF-specific treatments. These findings will plausibly make future interpretations of quantitative measurements of inflammatory biomarkers generalizable to sputum-producing patients in the CFFPR.

**Electronic supplementary material:**

The online version of this article (10.1186/s12874-019-0705-0) contains supplementary material, which is available to authorized users.

## Background

Neutrophil-predominant, intense airway inflammation associated with chronic airway infections drives CF disease. Multiple molecules [1–4] describe and mediate airway inflammation in CF. These biochemical markers of airway inflammation may mark disease progression and potentially provide surrogate markers of survival or intermediate outcomes that could improve the power of clinical trials to demonstrate efficacy of novel treatments [2, 3, 5–8]. Lung function, pulmonary exacerbations and weight are the best clinical predictors of survival [9, 10] and are the key end-points for multiple trials with high impact on CF treatment [11–17]. However, the insensitivity of these clinical markers requires large trial enrollments and prolonged observation to ascertain effects [11, 13, 15, 18]. Understanding relationships between these clinical features of disease and the laboratory measurements of biomarkers of inflammation will improve our knowledge of pathophysiology and enable better understanding of the impacts of current and investigational treatments.

Most evaluated inflammatory signals are increased in CF airways relative to normal airways [3, 19–21]. Three potentially useful biomarkers are associated with disease progression. High mobility group box-1 protein (HMGB-1), a highly conserved protein [22, 23], is associated with pulmonary exacerbations [24]. Sputum concentration correlates with concurrent lung function and number of prior-year exacerbations [25]. HMGB-1 sputum concentration predicts time to next exacerbation, number of subsequent pulmonary exacerbations and time to death or lung transplantation in CF. [25] Calprotectin, a proinflammatory molecule found abundantly in CF sputum, decreases with antibiotic treatment; high levels are associated with more rapid recurrence of a pulmonary exacerbation [26, 27]. Neutrophil elastase activity (NE) appears in airway secretions in CF [28], induces interleukin-(IL)-8 production [29], and predicts accelerated lung function loss in children with CF [5]. Finally, granulocyte macrophage colony stimulating factor (GMCSF) is another potentially useful biomarker. Its concentration in sputum at the beginning of hospitalization for a pulmonary exacerbation was strongly associated with the acute decrease in forced expiratory volume in 1 s (FEV_1_) normalized to percent predicted FEV_1_ for age, height, sex, race and ethnicity (FEV_1_%) [25, 30]. GMCSF may thus be an objective measurement of severity of an exacerbation.

However, all studies of these four potential biomarkers in CF were performed in single centers among patients recruited in ways subject to observer biases [5, 24, 25, 27]. Comparisons of predictive ability for future clinically relevant events between HMGB-1, calprotectin and neutrophil elastase cannot be done with existing data. We designed and implemented an observational study in the Mountain West CF Consortium (MWCFC) to allow direct comparison of the potential biomarkers and reduce biases. Because this study could facilitate the assessment of other clinically relevant conditions in CF and their relationship to inflammation with relatively small incremental costs, we included additional biomarkers and data collections in our study design to evaluate combinations of the primary molecules with each other and with other inflammatory markers to understand both our primary clinical event, pulmonary exacerbations, and additional secondary clinical conditions of high importance.

We report here the nature and implementation of our study design centered on randomized patient selection. This methodology strives to minimize observer bias in recruitment to maximize believability and generalizability of analysis [31, 32]. To demonstrate success, we compared cohort characteristics with patients in the CF Foundation Patient Registry (CFFPR) [33] in 2014 who would have fulfilled inclusion and exclusion criteria.

## Methods

### Study setting

Nine CF Care Centers accredited by the US CF Foundation and located in the Mountain West Region participated in this trial. The Mountain West Region includes (from North to South and West to East) Idaho, Montana, Wyoming, Nevada, Utah, Colorado, Arizona, and New Mexico. MWCFC centers provide care to about 10% of the patients with CF [34] who live in approximately 25% of the total land area of all 50 of the United States [35].

### Pre-study preparations

Prior to the general study planning meeting, the initial investigators (TGL, FRA, JLJ, RK, DRC) held multiple discussions to develop core statistical and logistical study plans. At the meeting, RK and DRC taught on core design issues (Additional files 1, 2 and 3, Video: Principles of Study Design: A Conversation Between David Cox and Ruth Keogh and Additional file 4: Transcript of Video) to improve the understanding of all investigators for study procedures. Two non-MWCFC collaborators provided guidance on additional important design elements (NL, ALQ), and three clinical investigators served as an external advisory committee (JPC, JSE, KNO) to review and modify procedures and provide oversight.


**Additional file 1:** Part 1 of the Video: Principles of Study Design includes overviews of Statistics and its meaning, the three main types of study design, and six principles that should guide design of studies. Total run time: 10 minutes 14 seconds. (M4V 342000 kb)



**Additional file 2:** Part 2 of the Video: Principles of Study Design includes a more in depth discussion of four of the six guiding principles of study design. Sir David and Ruth discuss that (1) studies should address interesting questions, (2) there should be a population of patients appropriate for the study questions, (3) measurements within the population should be done well, and (4) studies should avoid systematic error by including techniques such as balancing of patients and their characteristics and randomization with concealment to reduce observer bias in enrollment. Total run time: 8 minutes 17 seconds. (M4V 291000 kb)



**Additional file 3:** Part 3 of the Video: Principles of Study Design includes the two remaining points on study design. Sir David and Ruth discuss (5) that the number of individuals in a study should be sufficient to get good answers to the questions and (6) that the data collected should be capable of analysis and interpretation. The video concludes with a few remarks about unexpected problems during study performance and advice about the important aspects of study design. Total run time: 8 minutes 6 seconds. (M4V 292000 kb)


All personnel were trained at the University of Utah in study background, goals, inclusion and exclusion criteria, good clinical practice, patient safety and study procedures, data entry and records security. Research coordinators and laboratory personnel were trained individually at the University of Utah on sputum processing. The overall principal investigator (PI, TGL) and research manager (JLJ) performed on-site study initiation and training of investigators, study coordinators and laboratory technicians. Additional training was provided on-site or in Utah as requested. Training records were archived at each site and at the University of Utah.

### Study endpoints

The primary event for this observational trial is first pulmonary exacerbation following enrollment. The investigators at the general study planning meeting discussed previously published definitions for pulmonary exacerbations. Many clinical trials include a decision to start parenteral antibiotics in the criteria in order to facilitate retrospective identification of exacerbations [11]. After careful consideration, we decided to use the definition used in our original single center study (Table 1) [25] for several reasons: (A) we intended our primary event to be determined in a prospective manner, (B) a definition including use of parenteral antibiotics could have introduced bias in determination of a pulmonary exacerbation at the point of care, (C) the requirement for antibiotics might have been viewed as requiring an intervention despite ours being an observational study, and (D) a central goal was replication of results from the original single center study [25].

**Table 1:** Symptoms and signs of a pulmonary exacerbation of CF^a^

Symptoms	Signs
increased sputum, cough, dyspnea	10% drop in FEV_1_ or forced vital capacity
chest pain or tightness	Temperature > 38.4 °C
hemoptysis	Witnessed hemoptysis greater than 100 ml per episode
fever	SaO_2_ < 90% or PaO_2_ < 60 mmHg despite usual oxygen
chills	For adolescents a drop in SaO_2_ of 5% (for example, 97 to 92%)
arthralgias	Increased supplemental oxygen requirements
fatigue	Unplanned weight loss ≥5% of baseline body weight over 3 months
Other Considerations	
Respiratory arrest or failure requiring mechanical ventilation regardless of other criteria At site PI discretion for borderline cases or for cases with serious findings not included here	

^a^A pulmonary exacerbation is defined as the presence of one symptom and one objective finding

We considered using a scoring system to diagnose a pulmonary exacerbation [36] but felt that the additional complexity might reduce reporting of events especially for patients evaluated by clinicians not participating directly in the study. Because patients can be unstable but not quite reach the severity of disease described by our definition (Table 1), we defined a category of “mild exacerbation” to reduce the likelihood of arbitrary misclassification and to allow future investigation of this poorly defined clinical state.

The investigators and the external advisory committee reached unanimous consensus agreement with the definitions prior to enrollment of patients. These definitions were included in the application for investigational review board (IRB) or equivalent ethical oversight organization at all participating centers in the MWCFC.

Because there are multiple clinical questions for which insufficient information exists to allow independent studies, we discussed potential additional data collections and laboratory assessments. We incorporated additional clinical annotations and sample analyses that were judged feasible and that would have minimal potential to introduce bias or alter enrollment in our primary study.

### Enrollment

After obtaining IRB approvals, we reviewed center-specific potential participant lists. Each center identified patients with confirmed CF either (A) older than 18 years of age able to provide informed consent and to expectorate sputum or (B) older than 12 (but less than 18) years able to provide assent and able to expectorate sputum or successfully undergo sputum induction as part of normal care, with parents or guardians able to consent. We enrolled patients after written informed consent only when judged clinically stable by enrolling investigators. We excluded patients unable or unwilling to provide sputum or who had previously received organ transplantation, were on immunosuppression beyond oral prednisone or on treatment with immunologic-based biologic therapies. We excluded pregnant women, prisoners and other vulnerable patients from enrollment but did not require withdrawal if a patient became vulnerable because of the non-coercive nature of continued participation in an observational study. Patients, guardians or investigators could request withdrawal at any time.

### Power calculations

We sampled prior data and bootstrapped models to estimate the number of patients required to detect associations for HMGB-1 concentrations, our primary biomarker for study, with pulmonary exacerbations and survival outcomes [25, 37]. We additionally calculated the number of patients needed to study associations between GMCSF concentration and acute drops in FEV_1_% at the beginning of a pulmonary exacerbation [25] and NE with drops in FEV_1_% among children [5] over two years of follow up (Table 2). Based on these calculations, we planned a minimum enrollment of at least 40 patients to explore the ability of our primary biomarker, HMGB-1, to predict our primary event, time to first pulmonary exacerbation. However, we hoped to enroll as many as 125 to a maximum of 175 patients, including both adults and children, to enable a believable and financially feasible analysis (see Additional files 1, 2, 3 and 4) of GMCSF as a reporter of severity of a pulmonary exacerbation [25].

**Table 2:** Bootstrapped Power Calculations^a^

Row	Statistical Model	Outcome Variable	Biomarker, Concentration or Activity	Percent Power	α-Level	Estimate of Patients Required
1	Proportional Hazards	Time to first Pulmonary Exacerbation	HMGB-1	90	0.01	40
2	Proportional Hazards	Time to Lung Transplantation or Death	HMGB-1	90	0.01	30
3	Linear Regression	Acute FEV_1_% Drop with onset of a Pulmonary Exacerbation	GMCSF	80	0.01	175
4	Linear Regression	Acute FEV_1_% Drop with onset of a Pulmonary Exacerbation	GMCSF	85	0.05	125
5	Linear Regression	FEV_1_% Drop over 2 Years	NE	80	0.05	32

^a^Based on prior published results, we sampled patients and bootstrapped statistical models of predictor biomarkers for outcome variables, setting percent power and α-level in order to estimate the number of patients similar to prior patients needed to detect associations. See the study protocol for more details (Additional file 5)

### Randomized selection of patients

To minimize observer bias (Additional files 2 and 4) [31, 32] and enroll a representative sample of patients with CF, we assigned a randomly chosen letter of the alphabet to each potentially eligible patient using one adult and one pediatric block per MWCFC center. Based on a prior recruitment rate of 53% [25], and allowing for a 10% clinic no-show rate, we chose a letter of the alphabet to assign to each adult and pediatric center to serve as the threshold between random inclusion or exclusion from enrollment. We recruited eligible patients attending clinic who had been assigned personal random letters earlier in the alphabet than the threshold letter for the center. The method eliminated the need for research coordinators to visit clinic for patients with assigned letters later in the alphabet than the center’s threshold letter and allowed pre-clinic notification of potential enrollments and sample collections to clinical and laboratory personnel.

To maintain proportional enrollment among centers and reduce seasonal biases, we monitored enrollment rates at each center and adjusted the threshold letter earlier in the alphabet for enrollment that was too rapid and later in the alphabet for enrollment that was too slow after the first month and quarterly thereafter beginning with study month three. This enrollment speed adjustment strategy maintained randomization.

We required clinical stability and a sputum sample at enrollment. We defined clinical stability as the absence of a pulmonary exacerbation (Table 1) [25]. Because biomarker values change with time following an exacerbation, we collected the dates of the five exacerbations (if any) prior to the study enrollment date to enable adjustment for duration of clinical stability prior to sample collection. Guidance and examples were presented at the general study planning meeting and in the study protocol (Additional file 5, Study Protocol). Site PIs adjudicated borderline cases.

### Shipping controls

Prior to finalizing sputum handling protocols, we examined the effects of shipping prior to initial sample processing on HMGB-1 measurements. We recruited 10 adult patients and collected expectorated sputum in Utah on a single day after informed written consent. We divided samples in two and processed half immediately to isolate an aqueous fraction (see next section or Additional files 6 and 7 for specific details). We added protease inhibitor cocktail (Sigma, St. Louis, MO, USA) and froze at − 70 °C until ready to perform HMGB-1 assay. We kept the second half of each sample on ice and shipped to ourselves overnight at 4 °C (Fedex, Memphis, TN, USA), isolated the aqueous fraction, added protease inhibitor and froze. We measured HMGB-1 levels in both immediately-processed and shipped samples by ELISA using commercially-available antibodies (R&D Systems, Inc., Minneapolis, MN) [25, 38]. Results (see below) prompted incorporation of on-site sputum processing at each center.


**Additional file 7:** Video: Cystic Fibrosis Biomarker Study: Sputum Collection and Processing. The research team at the University of Utah demonstrate supplies and equipment needed to collect and process sputum specimens for the biomarker study. Total run time: 10 minutes 52 seconds. (M4V 105000 kb)


### Sputum collection, processing and shipping

We collected sputum after expectoration. We encouraged collection by alternating between research and clinical sample vials to minimize the disturbance of collection for cultures, but some research samples were collected after clinical samples [39]. We allowed sputum induction where that method was part of usual pediatric care. In accordance with Therapeutic Development Network standard operating procedure [40], we allowed one hour to collect sputum, transport from collection point on ice and initiate laboratory processing with an additional 1–3 h to complete processing. We further specified that during the initial hour, collection time was limited to 20 min, and 40 min were allowed to transport specimens on ice and initiate laboratory processing.

Samples were collected in 50 ml conical tubes supplied by the central study coordinators at the University of Utah. Following collection, samples were placed on ice and transported to the local processing laboratory. Sputum samples were weighed, diluted 1:1 with Hanks Buffered Saline Solution (HBSS, Sigma, St. Louis, MO) and vortex mixed for 1 min. A sterile disposable pipette was used to transfer 0.25 ml of the vortex mixed sample to a 1.8 ml tube containing Streck Solution (Streck Inc. La Vista, NE); contents were mixed using the pipette, and the tube was sent via Fedex (Memphis, TN) to the U of Utah for cell counts and differentials. The remainder of the vortex mixed sputum sample was centrifuged for 20 min at 2800 g at 4 °C to produce a top lipid layer, a middle aqueous layer and a bottom pellet layer. The layers were carefully separated by transfer pipettes; all personnel were trained to separate the layers in a way to avoid contamination of the aqueous fraction by the other two layers.

The lipid layer was carefully transferred to a new 1.8 ml cryovial, labeled with the participant study identification number (ID), collection date, fraction identifier (L) and sample number. The aqueous layer was divided in two. The first half was diluted with HBSS (Sigma) 1:1, vortex mixed for 10 s, aliquoted and labeled with participant study ID, date, fraction identifier (SA), sample number and aliquot number. The second half was diluted with protease inhibitor cocktail (Sigma) 1:1, vortex mixed for 10 s, aliquoted and labeled with participant study ID, date, fraction identifier (SB), sample number and aliquot number. The pellet was transferred to a 1.8 ml cryovial and labeled with participant study ID, date, fraction identifier (P), sample number and aliquot number. (See Additional files 6 and 7 for illustrated instructions for sample processing.)

Fractionated samples were frozen and stored at − 70 °C until shipping. Frozen samples were shipped on dry ice in split batches to prevent loss of any single sample (see Additional file 6) and immediately stored at − 70 °C in Utah until laboratory analysis.

We collected multiple samples from participants to address secondary study endpoints. To understand changes in airway inflammation, we collected sputum within 48 h of diagnosis of the first pulmonary exacerbation following enrollment and a convalescent sputum within 4–12 weeks of exacerbation onset. We encouraged collection of additional pulmonary exacerbation samples, particularly if a sample was missed at the time of first pulmonary exacerbation following enrollment. A final sample was requested at the end of the study whether the patient was acutely ill or stable.

A laboratory flow sheet with illustrated processing instructions was completed for each sample to ensure standardized processing and enable monitoring of individual samples (Additional file 6, Sputum Processing Instructions and Form). Written and video sputum processing instructions were available throughout the study (Additional file 5 and Additional file 7, Video: Cystic Fibrosis Biomarker Study: Sputum Collection and Processing).

### Clinical annotation

Research coordinators collected and recorded information simultaneously with sputum sample collections including demographics, CFFPR identification numbers, home address, number and dates of CF exacerbations in the year prior to enrollment, other lung disease, smoking, menstrual and pregnancy histories, treatments, physical exam findings, spirometry results, bacterial and alcohol and acid-fast culture results and questionnaire results including Borg dyspnea index [41], GAD-7 anxiety scale [42, 43], PHQ-8 depression scale [43, 44], brief pain index [45], selected Munich Chronotype Questionnaire [46] items (MCQ, written permission for use obtained from Dr. Roenneberg) and food insecurity [47], and dates of death or lung transplantation. Many of these data addressed exploratory endpoints deemed interesting and feasible and unlikely to bias enrollments by the investigators (Table 3). All data were stored using the Research Electronic Data Capture (REDCap) system [48].

**Table 3:** Data Collections for Exploratory Endpoints

Endpoints potentially related to inflammation	Pertinent Clinical Data Collected
Depression, Anxiety, Pain	PHQ-8 Depression Scale GAD-7 Anxiety scale Borg Dyspnea Index Brief Pain Index
Environmental Factors: Altitude, Air Pollution, Climate	Sputum Collection Date Home Address (Latitude, Longitude, Altitude) Microbiological Sputum Culture Results
Infection Status	Sputum Culture results
Sleep and Circadian Rhythm: Sleep Phase and Duration	Munich Chronotype Questionnaire
Menstrual Cycle	Last Menstrual Period Date
Food Insecurity	History of missed or potentially missed meals GAD-7 anxiety scale Food Availability focused questions

We reviewed REDCap and CF Foundation Patient Registry data for enrollment years to identify inconsistencies in study data. Study coordinators from Utah performed interim and end-of-study monitoring on-site to compare entered data with electronic medical records for every participant. Discrepancies were resolved by site study personnel prior to analyses.

### Statistical analysis

We use the R statistical system [49]. We used patient home addresses and *The National Map* [50] to derive longitude, latitude and elevation. We calculated GAD-7 and PHQ-8 total scores [42, 44], weight-for-age *z*-score [9], FEV_1_% [30, 51], and 5-year predicted survival [9]. We used χ^2^, *t*-test, linear regression and quasi-Poisson regression as appropriate to compare MWCFC with CFFPR data adjusted for study site, age, sex and other patient characteristics. We tested analyses for sensitivity to CF-specific treatments.

## Results

### Shipping controls

**Fig. 1:**
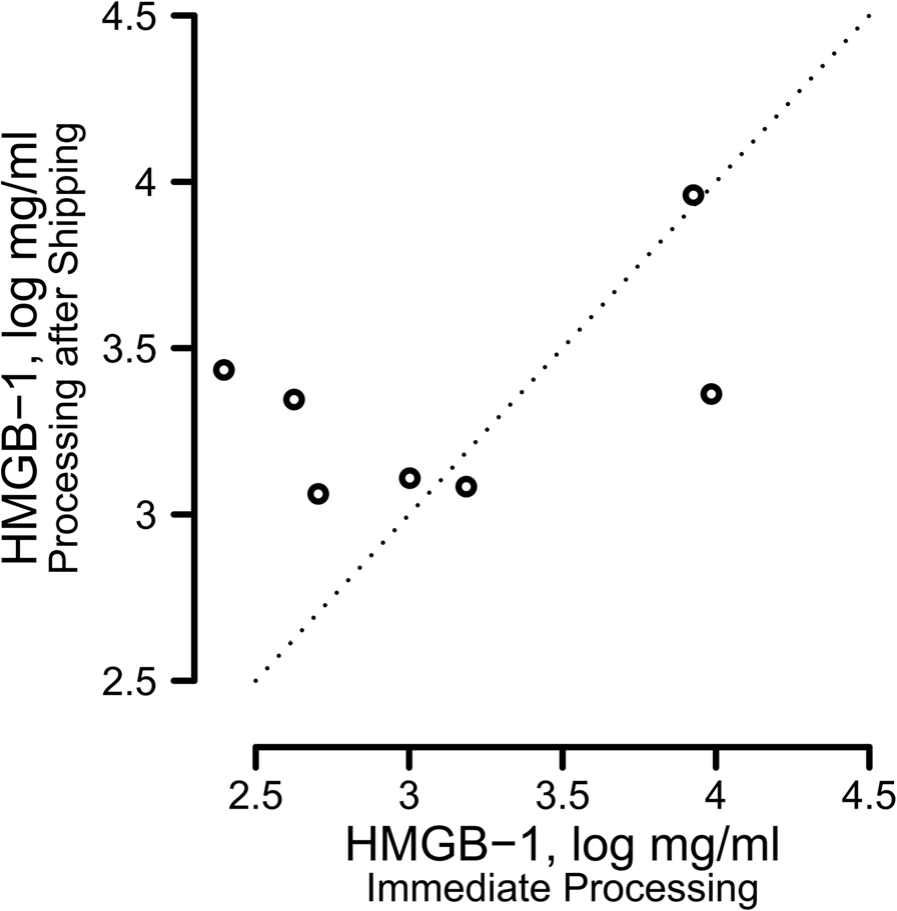
Shipping Effects on HMGB-1 Measurements. Overnight shipping of refrigerated unfractionated sputum samples from seven patients was associated with a statistically significant increase in ELISA measurements of HMGB-1, especially for low values. Although the values were correlated with values from samples that were fractionated and frozen prior to shipping (see text), the large and somewhat unpredictable sizes of differences in values and the compressed range of values overall suggested that on-site processing of samples would reduce measurement errors and better enable analyses involving HMGB-1

Among 10 volunteers, 7 produced sufficient sputum for evaluation. After overnight shipping at 4 °C, HMGB-1 levels were elevated (linear regression: coefficient = 1.15, intercept = 5.19, *p* = 0.001) by factors as high as 2.8 (mean elevation factor 1.40, 95% CI = 0–2.8) compared to paired levels from sputum processed the same day as collection (Fig. 1).

### Patients

**Fig. 2:**
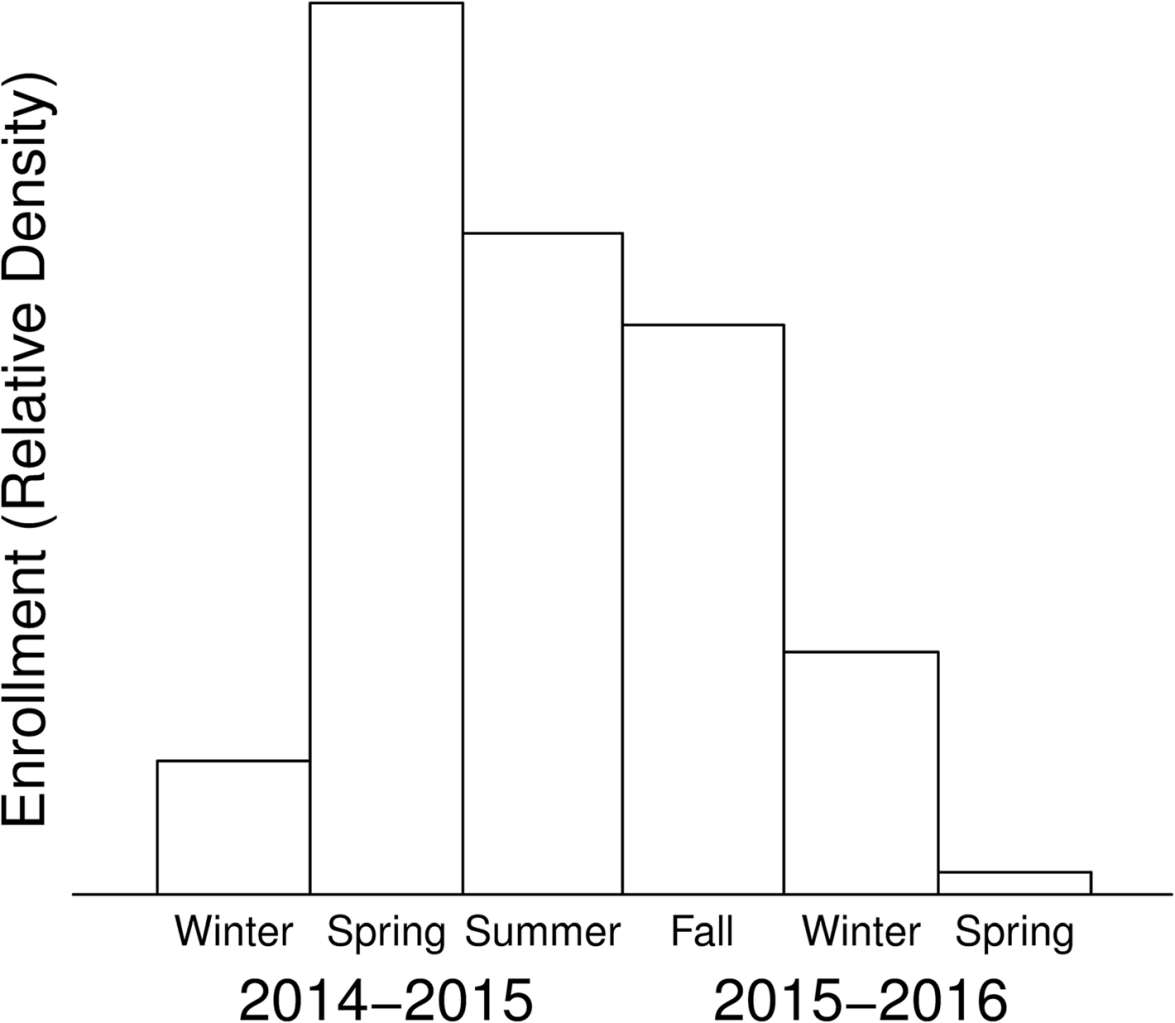
Patient Enrollment Distribution. The number of patients enrolled varied through the enrollment period of the study. Analyses demonstrated that there were no detectable seasonal biases introduced by differences in enrollments

Investigators enrolled 116 of the 154 children and 696 adults with confirmed CF in the MWCFC able to expectorate sputum; two patients were too young and were excluded after enrollment (Table 4). First enrollment was December 8, 2014; last enrollment was January 16, 2016 (Fig. 2). Five patients did not complete the sample collection portions of the study due to lung transplantation, death or other cause of loss of contact between study center and the patient. However, all 114 patients allowed long term follow up by providing permission to use their specific CF Foundation Patient Registry ID numbers. Queries specific to the study are ongoing for end-of-study data (Table 5).

**Table 4:** Comparison of Annualized CFFPR Data between MWCFC and other US Patients

Patient Characteristics	MWCFC, *n* = 114		CFFPR 2014 *n* = 14,394	*p*
	Enrollment	Annualized for 2014		
Male sex, fraction	0.46	0.46	0.48	0.79
Age, Years, mean (SD)	28 (12)	27 (12)	27 (12)	0.71
FEV_1_, Percent Predicted, mean (SD)	70 (22)	80 (23)	77 (27)	0.094
Height, cm, mean (SD)	167 (9.95)	167 (10.2)	166 (10)	0.39
Weight-for-age z-score, mean (SD)	−0.17 (0.98)	−0.20 (0.93)	−0.29 (1.1)	0.34
Pulmonary exacerbations in year prior to enrollment, median (range)	1 (0–7)	1 (0–6)	1 (0–14)	0.55
Pulmonary exacerbations in year prior to enrollment, mean (SD)	1.7 (1.7)	1 (1.2)	1.1 (1.5)	> 0.99
Patients with no pulmonary exacerbations, n (fraction affected)	79 (0.69)	63 (0.56)	7054 (0.52)	0.46
Diabetes, n (fraction affected)	25 (0.22)	26 (0.23)	4423 (0.31)	0.073
Pancreatic Sufficiency, n (fraction affected)	9 (0.079)	19 (0.17)	2239 (0.16)	0.84
CF related arthropathy, n (fraction affected)	8 (0.07)	10 (0.088)	697 (0.048)	0.085
5-Year Predicted Survival, median (range)	0.959 (0.094 to > 0.999)	0.973 (0.464–0.999)	0.967 (0.0949–0.999)	0.17
Home Altitude, m, mean (SD)	1305 (442)	–	–	–
Infections Present, n (fraction affected)				
Methicillin Sensitive *S aureus*	51 (0.45)	67 (0.59)	7937 (0.55)	0.5
Methicillin Resistant *S aureus*	21 (0.18)	33 (0.29)	5001 (0.35)	0.23
*P aeruginosa*	70 (0.61)	84 (0.74)	10,096 (0.70)	0.47
*B cepacia* complex	3 (0.026)	5 (0.044)	782 (0.054)	0.78
*S maltophilia*	7 (0.061)	26 (0.23)	2970 (0.21)	0.65
*Achromobacter* spp	5 (0.044)	11 (0.096)	1579 (0.11)	0.76
*Candida* spp	17 (0.15)	17 (0.15)	3100 (0.22)	0.11
*Aspergillus*	12 (0.11)	26 (0.23)	3091 (0.21)	0.82
*Mycobacterium avium-intracellulare* complex^a^	1 (0.0088)	8 (0.082)	730 (0.069)	0.74
*Mycobacterium abscessus*^a^	3 (0.027)	5 (0.052)	527 (0.05)	> 0.99
Treatments in use, n (fraction affected)				
Any Form of Inhaled Tobramycin	38 (0.33)	71 (0.62)	8069 (0.58)	0.38
Inhaled Aztreonam	40 (0.35)	47 (0.41)	4977 (0.36)	0.25
Oral Azithromycin	62 (0.54)	67 (0.59)	8595 (0.60)	0.91
Inhaled Hypertonic Saline	71 (0.62)	82 (0.72)	9611 (0.69)	0.54
Inhaled DNase	101 (0.89)	106 (0.93)	12,168 (0.87)	0.087

^a^Fractions reported reflect that 97 MWCFC and 10,618 CFFPR patients had acid fast bacterial cultures performed in 2014. MWCFC patients were more likely to undergo acid fast cultures than non-study patients (*p* = 0.008, χ-square test)

**Table 5:** Collections

Collection Type	Enrollment	First Pulmonary Exacerbation Onset^a^	First Pulmonary Exacerbation Convalescence	Additional Exacerbation	End of Study Follow Up^b^
Clinical Data	114	92	36	10	72
Samples	114^c^	52	29	8	62

^a^Follow up varied and sometimes exceeded one year to the first exacerbation. Among enrolled patients, 81% had an exacerbation during the study. However, the observed percentage of patients with exacerbations within 1 year was lower, 47%, and was similar to the 44% reported in annualized 2014 CFFPR data for this cohort of patients (Table 4)

^b^Queries for data from the end-of-study are ongoing at the time of submission

^c^114 samples were collected, however, only 112 were sufficient in size to allow laboratory analyses

Enrollment ranged by study site from 0 to 43% and 1 to 28% of eligible children and adults, respectively. The proportion of enrollment was unrelated to center size for pediatric, adult or combined age groups (linear regression, *p* > 0.1, all groups). Using linear, logistic or quasi-Poisson regression as appropriate for the outcome variable assessed, we evaluated seasonal effects on enrollment. Relative to Summer enrollment, patients enrolled in the Fall were 8.7 years older (*p* = 0.02) while patients enrolled during the Winter had an FEV_1_% 15.9 percentage points higher (*p* = 0.004). There were no significant relationships for sex, weight-for-age or height-for-age [52] *z*-scores, number of prior year pulmonary exacerbations, diabetes, pancreatic sufficiency, 5-year predicted survival [9], anxiety or depression scores, any infection status or patient home altitude. Despite statistically significant findings for characteristics considered individually, no seasonal differences remained significant after Bonferroni correction.

### Comparison of patient characteristics with National Data

The 2014 CFFPR reports 14,394 patients able to expectorate sputum, 12 years of age or older. We found no significant differences in disease characteristics, microbiology results or prescribed treatments compared with MWCFC patients (Table 4).

### Sputum samples

Two shipments of samples (out of approximately 30 total shipments to date for the study) arrived in Utah partially thawed despite verified adherence to shipping procedures and appropriate packing with dry ice at the two originating centers. However, because we required portions of every sample to be split at the time of on-site processing as safeguards against shipping problems, unthawed aliquots of every sample collected were available for laboratory analyses.

The natural log of the average cell count per milliliter was 16.2 (SE 8.11) based on 112 of 114 enrollment samples of sufficient volume without oral contamination defined as ≥5 squamous epithelial cells per low power light microscopy field on modified Wright’s stain (see Additional file 5). The average differential of 36% neutrophils, 30% lymphocytes, < 1% eosinophils and 34% other cells was based on 108 out of 112 samples. A few samples had inadequate preservation in transport or sparse cellularity thus less than 200 cells were counted, but we counted 200–500 cells for the rest of the samples in accordance with Therapeutic Development Network standard operating procedures for samples from patients with CF collected for inflammatory marker measurements (mean 250 cells/sample, range 41–541) [40]. Total processing time from initiation of sputum collection to completion of processing was a mean of 54 min (SD = 1 h 38 min, max time = 3 h 45 min).

A total of 267 sputum samples were collected from patients. There were 116 enrollment samples, but two of those patients were excluded because they were too young to participate. The primary outcome for the study is time-to-first pulmonary exacerbation, and we captured the time-to-event data for the 92 patients who had these events during follow up.

To address secondary outcomes, we successfully collected additional data at the time of the first exacerbation during study follow up for 92 of the 114 enrolled patients and collected sputum samples for 52 of the events. Two additional sputum samples were collected in patients with subsequent pulmonary exacerbations where the first exacerbation sample was missed. The major reasons for missing first exacerbation sputum samples were diagnosis away from the CF Care Center and lack of ability to produce sputum within the 48 h collection window.

## Discussion

We implemented a design that focused on randomized patient selection for a multicenter observational study of inflammatory sputum biomarkers in CF. We achieved our primary enrollment goal of recruiting a cohort representative of patients in the US with confirmed CF, 12 years of age or older and able to produce sputum. Age, sex, lung function, growth and nutrition factors, major morbidities including pancreatic insufficiency, CF related diabetes and airway infection prevalence and treatment frequencies were similar between MWCFC and national patients (Table 4). We achieved our secondary goal of minimizing seasonal and care-center size effects on patient selection. Randomization reduces observer-dependent selection bias and increases believability of data collection, generalizability of results and security of interpretation (Additional files 1, 2, 3 and 4) [31, 32].

Our investigation of the effect of shipping on unprocessed samples found a potential source of inaccuracy for HMGB-1 measurements (Additional files 3 and 4). Overnight delay in processing was associated with large increases in measured concentration for low values found after same-day sputum processing. Conversely, we found decreased values for samples processed after overnight shipping on ice that had high measurement results from same day processing. Together these effects appeared to compress the range of values, potentially reducing our ability to discriminate between patients with similar levels of inflammation (Fig. 1). These observations coupled with our intention of reproducing prior findings derived from same-day processing of sputum compelled us to require on-site processing for the current study. Shipping and central processing of sputum might similarly affect other potential biomarker measurements, but we did not specifically address this question.

Our method of sputum sample processing repeats the method we used to discover associations between HMGB-1 and pulmonary exacerbations and survival [25]. In that effort, we modified the standard operating procedures of the Therapeutic Development Network [40] by avoiding the use of dithiothreitol (DTT) in order to optimize detection of some inflammatory biomarkers [53]. Others studies of the effects of avoiding DTT and adding protease inhibitors found that biomarker detection was improved in both cases [53–56].

Despite the care taken, our study has limitations. We originally planned to enroll up to 175 patients (Table 2, row 3) based on the analysis of numbers of patients needed to replicate our prior results with GMCSF with a high level of precision to answer our research question (Additional files 1 and 4) [25] about the relationship between GMCSF and acute FEV_1_% drop with onset of a pulmonary exacerbation, a secondary goal. Unfortunately, we fell short of the planned number; however, the actual number of patients enrolled still allows us to address this question (Table 2, row 4) and well exceeded the estimated numbers of adult and pediatric patients to replicate prior results that revealed relationships between HMGB-1 and pulmonary exacerbations, our primary goal. We additionally achieved recruitment numbers to provide sufficient power to analyze secondary outcomes including relationships between HMGB-1 and NE with pulmonary exacerbations, subsequent lung function and combined lung transplant and death outcomes (Table 2, rows 1, 2 and 5).

Because the study was non-interventional, we did not include the final question of the PHQ-9 regarding specific plans to commit suicide, because positive answers would have required intervention and a different regulatory approval pathway. Instead, we alerted clinicians to patients with high PHQ-8 scores and requested them to consider intervention as part of usual clinical care; this plan was specifically approved by all site IRB’s.

We attempted to reduce bias against enrollment of patients that frequently miss clinic appointments. However, the nature of clinic non-adherence likely still reduced enrollment of these patients and may have reduced our ability to collect subsequent samples and data for our secondary study goals.

We successfully collected samples for only 52 out of 92 patients who suffered pulmonary exacerbations during study follow up. The high degree of missingness suggests that there may be hidden biases in studies of pulmonary exacerbation. Because most samples were missed due to diagnosis at non-participating care centers, distance-to-center or other reasons that impede care at an accredited CF Care Center may be previously unrecognized sources of bias in studies of pulmonary exacerbation. Further analyses of the current data and additional future studies designed to collect sputum from outlying care centers may help to understand the nature, size and effect of such bias.

Our study did not enroll patients with the same clinical status as in prior studies [5, 24, 25, 27]. Prior patients were non-randomly selected often after or during a pulmonary exacerbation. We reasoned that predictions from biomarkers measured during clinical stability would be most generally useful, thus our current study patients may be less ill as a group than previously reported patients.

## Conclusions

We designed and implemented an observational study of stable patients with CF able to produce sputum to identify inflammatory biomarkers predictive of clinically important outcomes. Our design and enrollment efforts recruited a well characterized cohort from the MWCFC region similar to sputum-producing patients throughout the US that participate in the CFFPR. This cohort provides a believable and generalizable foundation in sufficient numbers for clinical interpretation of upcoming analyses of biochemical marker measurements from the carefully collected and annotated sputum samples.

## Additional files

Additional file 4: Transcript of Video: Principles of Study Design: A Conversation Between David Cox and Ruth Keogh. (PDF 124 kb)

Additional file 5: Final Study Protocol. Created and distributed 13 May 2013. (PDF 290 kb)

Additional file 6: Sputum Processing Instructions. Provides the laboratory flow sheet for sample collection and illustrated instructions. (PDF 706 kb)

## Abbreviations

CF: Cystic Fibrosis
CFF: Cystic Fibrosis Foundation
CFFPR: Cystic Fibrosis Foundation Patient Registry
ELISA: Enzyme Linked Immunosorbent Assay
FEV_1_: Forced Expiratory Volume in one second
FEV_1_%: Percent Predicted FEV_1_
GAD: Generalized Anxiety Disorder
GMCSF: Granulocyte-Macrophage Colony Stimulating Factor
HBSS: Hanks Buffered Saline Solution
HMGB-1: High Mobility Group Box-1 protein
IL: Interleukin
IRB: Investigational Review Board
MCQ: Munich Chronotype Questionnaire
MRSA: Methicillin Resistant *Staphylococcus aureus*
MWCFC: Mountain West Cystic Fibrosis Consortium
NE: Neutrophil Elastase
PHQ: Patient Health Questionnaire
REDCap: Research Electronic Data Capture
SD: Standard Deviation
