# Supplementary material for: Prospective multicenter randomized patient recruitment and sample collection to enable future measurements of sputum biomarkers of inflammation in an observational study of cystic fibrosis
Source: BMC Med Res Methodol. 2019 Apr 26;19:88. doi: 10.1186/s12874-019-0705-0 (PMC6485181; doi:10.1186/s12874-019-0705-0)
Supplement: Supplementary file 5 — Transcript of Video: Principles of Study Design: A Conversation Between David Cox and Ruth Keogh. (PDF 124 kb) [file 12874_2019_705_MOESM4_ESM.pdf]

e-Appendix 1. Transcript of Video 1. Principles of Study Design: A conversation between David Cox and Ruth Keogh.

David Cox: Ruth, it's interesting isn't it that Statistics means such different things in different contexts. In everyday life and indeed historically, it essentially meant counting heads, census, how many people died from this, died from that, how many people are employed, right through all things about everyday life, even sport. For example, take the very important topic that there are statistics of all important cricket matches played in England since 1864. No doubt other games in Sports have similar things.

But when we go to Science, in some contexts, it means *p*-values, passports to publication. If your *p*-value is 0.049, "Hooray, we can publish!" If it's 0.051, "Disaster." Most statisticians, perhaps all statisticians, would take that rather disapprovingly, to put it mildly.

Ruth Keogh: I guess it's the case, or would you say, a lot of work that's been done in particularly medical statistics and epidemiological fields has focused on complex methods of analysis from the standing of the complicated relationships between exposures and outcomes or between many variables.

David Cox: Yes, understanding complex situations if possible by doing simple analysis but, yes, that's the technically interesting part of statistics.

Ruth Keogh: A further aspect that perhaps has received less attention in the literature is the design of studies as opposed to the analysis.

David Cox: Yes indeed. So shall we talk about and concentrate on design in our conversation?

Ruth Keogh: Yes.

David Cox: I think we should begin by making some general points again, particularly, but by no means only, in a medical context.

We can think of three main types of study. The prospective, in which one starts with some patients, records some aspects of their nature and health, and then follows them forward to see what happens to them and hopefully explain the outcome in terms of what one observes. And in contrast to that, one can do a retrospective study particularly suitable for very rare outcomes where you start with patients who've had an unusual outcome, compare them with some controls and look backwards in time, again with the hope of understanding or explaining what they've experienced. And then there is a third type of study, the randomized clinical trial, which is distinguished, because it is an intervention. In observational studies, the observer observes and measures and records but doesn't actually necessarily immediately intervene in the patient's life. Whereas in the randomized clinical trial there are alternative regimes, and the patient is, in principle, randomized between them again with the objective of explaining the outcome that is ultimately observed.

Ruth Keogh: And the type of study that we use depends really on the setting that we are in, the exposures that we're interested in, depending on our particular interests. I suppose in principle, a lot of things could be investigated using any of these designs. It's just what becomes most convenient or most useful. So, one example is the relationship between smoking and lung cancer, which now is well established, of course. But the original study on that was a retrospective study done in the 1950's, the early 1950's, but there has also been a long term study of doctors in the UK. That was a prospective study in the UK. In theory, you could study the effect of smoking on lung cancer using an experiment

in which people are randomized to smoke cigarettes for a certain length of time and other people aren't, although the ethics of that would be debatable, of course.

David Cox: Yes (chuckle). And, of course, there have been many studies on animals of that issue.

But I think that at one level, the point of the randomized intervention is that the causal interpretation of the answer is much more secure. That if two groups have been randomized, in principle, any difference between them is either pure chance or the intervention. Whereas in observational study, there may be features that have not been observed that really, really account for what's going on.

I think the point that we should emphasize is that, from the design point of view, although these three types of study in the technical statistical literature turn to be treated entirely separately by very different people, nonetheless, if one stays away from the complex details and technicalities and concentrates on the basic issues, they are really common, and it is the commonality we would both want to emphasize in what we say.

Ruth Keogh: I think these common points don't necessarily involve lots of complicated mathematics, and the broad principles are essentially simple, even though in implementing them they might not be simple.

David Cox: No, in a sense, I think, in what we talk now we have the possibility of writing up formulae but I don't think we'd want to have any. There are no formulae in what we plan to say I think on this. And the difficulty is exactly what you say. In all fields of study, there's what we'd like to do when in principle it's to get a study with a secure conclusion. But there are always constraints, ethics, money, whatever. The scale in application is to, as far as possible, achieve the principles that from a common sense point of view are desirable and stick within the framework that practicality forces on us.

Ruth Keogh: So shall we talk about some of these main principles.

David Cox: Yes. I'm thinking about this like they can be organized in various ways. I think it would help to put them under 6 different headings, each of which could be discussed in detail. But we will discuss the six briefly.

The first is that the research question and the objectives of the study should be, of course, interesting but also well...reasonably, precisely defined.

The second is that the population of patients for inclusion in this study should be in some sense be appropriate ones.

The third one is about measurement, that the measurement technique should be suitable, and the appropriate variables recorded.

The fourth case (we now go towards conclusions), the fourth should be that there should be no suspicion of systematic error in the final conclusion.

The fifth is that the size of the study, number of patients, etc, should be sufficient to give conclusions that are reasonably incisive and indeed capable of answering the questions that one asked at the beginning.

And the sixth may seem a bit silly almost to say, but it is very important that the data should be capable of sensible analysis. There are plenty of examples around, probably not very many, but enough, to say that there, in completely relatively complex investigations, there is the danger of collecting data that can't be analyzed or can only be analyzed by making very strong assumptions.

[End of Part 1, Additional File 1, run time: 10:10]

Ruth Keogh: So shall we talk about these points in a bit more detail in turn?

David Cox: Yes, let's do that.

Ruth Keogh: The first point was about the fact that the research question should be interesting or well defined for the objectives of the study.

David Cox: Yes but there is also the feature that one of the basic statistical principles [is] that usually it is better if you have several questions. The root of the issue: study them one at a time or study them collectively in a single study. There are considerable advantages to studying questions collectively up to the point where [one is] trying to do too much in one study. The administration of the study will get difficult, complicated, and, perhaps more seriously, the quality of the measurements may get degraded if there is an attempt to measure too much.

Ruth Keogh: What would you say about situations which perhaps in recent years where we have very large data sets, banks of biological material, for example, where there's lots and lots of questions but not necessarily were asked in advance of collecting the data.

David Cox: Well, I suppose if people are clever enough to collect the data to address interesting questions, all to the good; and particularly if collecting enough data takes a long time that's a good way to proceed. Having been someone who started in physics labs, that's not the way I naturally think about problems. I tend to think myself perhaps old-fashionedly, "Let's have an incisive question...questions and then collect the data to analyze them." But different approaches are sensible in different contexts, I guess.

Ruth Keogh: The second point was about that we must have a population or should aim to have a population of patients for our study which is appropriate for the questions.

David Cox: Yes. Well, one basic principle there I suppose is that if one is beginning a totally new field without much background knowledge, it may be best to try and find a very uniform group of patients: possibly in a randomized trial, the use of twins so that the comparisons [are] between different twins or in an ophthalmological experiment between...using the two eyes as different individuals for comparison. But the more one goes towards getting more partly a subject that is well investigated but also getting towards recommendations for immediate implementation and practice, the more the issue of the representativeness of the patient becomes more important, and so one would want a wide range of patients to test the dependence of any conclusions on ethnicity, age, etc., socioeconomic class, etc., so that there are contrasting tensions I think in that.

Ruth Keogh: So the third point moves on away from the observation of or catchment of participants on to what we measure in those participants and the importance that the things we measure should be appropriate for our questions and that they should be measured well or well enough for what we want to study.

David Cox: Yes and that is an enormous theme isn't it, one that we could spend some time on. A particular feature might be worth mentioning. Particularly in a study in a newish field is that of having a data audit at some point. What's the relation between the data as immediately recorded by whoever records it and what's on the computer. And it can be fruitful to have a quite independent person take a small sample of individuals near the start of a study and just check that there is a reasonable match between the two. It can't always be taken for granted.

Ruth Keogh: So this issue of measurement leads us onto the next point which is about systematic error....Various different sources of error could come from how we select the participants for the study. It could come what we measure on them and who measures it, and where it's measured and so on.

David Cox: Yes, there are many ways. And there are two techniques for dealing...as far as possible, avoiding systematic error where one is suspect. One is some notion of balancing that if there are two people doing measurements or assessing scan results or something of that sort that two treatment regimes being measured that each measurer should have half of each treatment, so to speak, so that you don't end up with a situation where the comparison of two treatments is in fact a comparison of two people or two sets of equipment or whatever it might be.

Then the other quite different aspect is randomization with concealment which is the technique for dealing with personal, subjective biases that can enter many different aspects of a study from choosing the patients to assessing the final state of the patients. And again, if...again if sensitive comparisons are involved, it is strongly preferable. There are certainly examples in the literature where experiments have been ruined by failure to do this. Measurers should not…that various aspects of the process should be concealed from the people making the choice whether it is choosing patients or assigning treatment groups or whatever. It may mean in some studies not just one act of randomization but randomization at every stage at which subjective errors might enter. And of course, some judgment has to be used over this, because if the errors involved are really, really known to be very small then a lot of fuss about randomization is not a good idea. It can cause more trouble. But in other context, not just randomization but randomization with concealment seems a very important thing to do in all these types of study.

Ruth Keogh: Randomization is normally associated with the experimental type.

David Cox: Well. It is mostly. It is the word used in the medical context. It is mostly used in the term randomized clinical trials...randomized controlled trials. Yes, that's right.

[End of Part 2, Additional File 2, run time 8:13]

Ruth Keogh: So the next point moves onto the size of the study and the fact that the number of individuals should be enough to get good answers to our questions. But I suppose at the same time it shouldn't be too many such that we've wasted time and other resources.

David Cox: Yes. This is normally expressed in terms of a prior calculation of the power of this study. I think a better way is to say what precision of estimation will you achieve at the end of whatever it is you are interested in, the slope of some relationship, the presence of some relationship, the difference between two treatment regimes or whatever it might be. And one should calculate either the number of patients that are necessary to achieve what seems a modestly sensible target. Or sometimes I think it is the other way around, really, that you have some rough assessment of how many patients are likely to

be available. Is this enough or indeed too many for the purpose that you want to avoid doing a study that is more or less doomed to be inept...or not inept but not precise enough to answer the question.

Ruth Keogh: I suppose the problem is that sometimes we're in a situation where we really don't know, it's exploratory, so that we really don't know what the kind of effect sizes are that we're going to see...

David Cox: ...or the variability that we're likely to encounter. A lot depends on whether this is a study in a field that has been explored at least to some extent beforehand in roughly similar investigations or whether it is something totally new. And if it is something totally new, there is the whole issue of the desirability and perhaps the necessity of a pilot study just to see how things work out. That's connected with the notion of a data audit as well.

Ruth Keogh. Yes. The final point, I don't know that you have anything to add on this, is that the data...it seems obvious, but you said that there are examples where this doesn't happen, that the data are capable of analysis and interpretation.

David Cox: Yes, well. There are a number of issues here. One is that we said at the beginning that the research objectives are clear at the beginning, well formulated at the beginning. In principle, that's highly desirable. But it shouldn't preclude the possibility that in the middle of the study something exciting and new comes up in which case one would have to do something that was not planned in advance.

I give a little example of that in a study I was involved in recently in which there were three treatments, two of which were expected to give beneficial results. After a while it became clear that one of these treatments was distinctly harmful. And it was important to under...not just to have detected that but to understand why this unexpected event happened. And on some thought, it was clear that there were two quite different explanations of this harmful event. And so a supplementary study had to be set up, on a very small scale admittedly, to resolve the dilemma of which of these explanations was right.

I think in principle that this can be a dangerous route to take that certainly in any study that goes on for a long time there should be at least a possibility, perhaps halfway through or something like that, of making changes, perhaps deciding that some possibilities are unimportant or some variables... some new variables need measuring or whatever it might be. It's dangerous to do that too often. It could cause total confusion.

Another issue...another similar issue might be that the number of patients accrued into the study was substantially less than expected. One might decide that the age range of patients in the protocol is too narrow – widen the age range a bit and take as from a precisely defined date and allow in the analysis for the possibility that these extra patients that you would then include are somehow different from the others or that there is some change in the study from that time point. In principle, you should have everything set up beautifully in advance, but I think to totally deny oneself the possibility of change could really be quite dangerous.

Ruth Keogh: So we shouldn't...our analysis, our design and what we do with optimization...

David Cox: Mostly design and then analysis...yes, I think so. And of course, I realize that regulatory agencies for perfectly good and understandable reasons tend to like things very well formulated in advance. And that's good.

Ruth Keogh: The cost and the timing of everything is not random...

David Cox: ...and the details of the analysis. And the answer to that is that one might have to do the analysis precisely as initially specified and then explain why it was inappropriate. But that's rather special.

We've talked entirely of simple ideas that are, one might say, are not statistical but common sense. But of course underlying what we're saying is a lot of specialized literature particularly on the design of experiments, but on the other studies as well, there is a complex set of mathematical theory and statistical design to enable us to study many features simultaneously, which are both extremely interesting and occasionally very useful though perhaps not so much in medical and epidemiological contexts. But I think the essence of what we're saying is that that's not the important aspect of study design.

Ruth Keogh: Well I think that some advice that you've given to me and that I've heard you give to other people on the nature of study design and the analysis is to keep it simple.

David Cox: Yes. I think that in all statistical work you should keep things as simple as possible but no simpler.

[End of Part 3, Additional File 3, run time: 8:02]

Video recorded 29 March 2013 at the London School of Hygiene and Tropical Medicine by Professor Sir David Cox, Honorary Fellow, Nuffield College, Oxford, UK and Ruth Keogh, Lecturer, London School of Hygiene and Tropical Medicine, London, UK.

Original Presentation 12 April 2013 at the MWCFC Meetings, Salt Lake City, UT, USA.

Combined Duration: 26 min 37 sec.

Transcript created 3-6 July 2018 by TG Liou.
