# Supplementary material for: Prospective multicenter randomized patient recruitment and sample collection to enable future measurements of sputum biomarkers of inflammation in an observational study of cystic fibrosis
Source: BMC Med Res Methodol. 2019 Apr 26;19:88. doi: 10.1186/s12874-019-0705-0 (PMC6485181; doi:10.1186/s12874-019-0705-0)
Supplement: Supplementary file 6 — Final Study Protocol. Created and distributed 13 May 2013. (PDF 290 kb) [file 12874_2019_705_MOESM5_ESM.pdf]

e-Appendix 2. Final Study Protocol, 13 May 2013.

Background and Significance

Survivorship in CF

The initial description of CF presented patients dying at or shortly after birth due to GI catastrophes or rapid starvation due to combined fat and protein malabsorption.[1] The development of surgical techniques to address meconium ileus, intussusception, volvulus and other related causes of small bowel obstruction along with the advent of pancreatic enzyme replacement shifted the primary cause of death from GI to pulmonary disease.[2] Multiple treatments in addition to pancreatic enzyme therapy led to increasing survival including treatment of pulmonary infections with antibiotics, optimization of other pulmonary therapies, organization of specialized care centers, persistent careful attention to nutrition.[3,4] All survivorship studies since the introduction of pancreatic enzyme therapy that identify causes of early death in CF identify declining lung function as the key factor,[5–11] and most of these studies found that increasing APE frequency associated with chronic airway infections was the second major factor leading to early death in CF. Pancreatic enzyme therapy rendered malnutrition a consistent but lesser contributor to worsened survival.

The overall effect of improving treatments has been a remarkable improvement in survival in CF. Current median age at death in the United States due to CF has risen to approximately 26 years of age while predicted median survival has hovered for several years in the range of 35-40.[12] Nevertheless, these ages are less than half the expectation of life at birth for Americans.[13]

Airway Inflammation in CF

Chronic infection due to multiple organisms, such as *Pseudomonas aeruginosa*,[4,14–16] drives lung disease in CF by creating intense but dysfunctional neutrophil-predominant airway inflammation involving mucus plugging, respiratory epithelial cell necrosis and destruction of structural lung components.[17] Acute pulmonary exacerbations (APE) punctuate the clinical course and require hospitalizations for intensive treatment. Improvements in therapy over seven decades include improved broad spectrum antibiotics,[4] mucolysis,[18,19] *P aeruginosa* suppression with chronic inhaled antibiotic,[20,21] and suppression of inflammation.[4,22] Each new therapy incrementally extended survivorship,[23] but the expanding list of concomitantly administered therapies creates treatment burdens that consume four or more hours daily.[3,23] Yet, these treatments only slow and do not stop gradual or stepwise loss of lung function.[4] Patients require refined or new therapies with reduced treatment burdens to improve survival and quality-of-life.[3,23]

Lung Function and APE Measurements in the Development of Therapies for CF

Therapeutic trials for CF performed over the last 40 years have consistently used pulmonary and APE-associated measurements as their major outcomes because these are the two most heavily weighted predictors and therefore surrogates of survival.[10] Alternate day steroid,[24] antibiotics and chest physiotherapy,[25] recombinant human DNase,[18] inhaled tobramycin,[20] hypertonic saline,[19] inhaled aztreonam[26] and ivacaftor[27] all improved lung function and most also reduced APE. Some additional studies found no lung function effect, possibly due to enrollment or follow up time limitations. Use of combination antibiotics rather than a single β-lactam for APE treatment lengthened time to the first subsequent APE[28] while chronic oral azithromycin reduced APE over time.[22,29] None of these therapies were able to show a change in survival due to the number of patients and limited observation times.

Lung function and APE-associated outcomes vary on a year-to-year basis requiring trials either to continue for extended periods or enroll large numbers of patients to overcome imprecision in reporting therapeutic impacts. Biologically relevant, accurate, reproducible, sensitive and specific biomarkers relating airway inflammation to lung function and frequent APE would energize the assessment of proposed new treatments.[30] A biomarker or biomarker panel that provided prospective, statistically significant predictions of future lung function, APE and especially survival would be a superb surrogate for clinical outcomes of the greatest relevance for evaluating new therapies in CF.

Potential Biomarkers in CF

The inflammation in CF airways is not present in healthy controls, thus biomarker candidacy begins with abnormal increases or decreases in biomarker levels compared with normal. Interleukin-(IL)-1β, IL-6, IL-8, mannose-binding lectin and TNF-α are elevated in CF while IL-10 is reduced.[31,32] Production of IL-2 is increased and IL-8 is increased in circulating T-cells in CF.[33] IL-3, IL-5, regulated on activation, normal T-cell secreted (RANTES) and GM-CSF were differentially expressed in CF patients compared to asthma and acute bacterial pneumonia patients.[34]

Biomarkers associated with key features of disease status represent candidates for being causal agents. Bacterial infections are associated with worsening lung function, and lung function responds to antibiotic therapy,[25,35–37] however decline in lung function seems more specifically due to the inflammatory responses.[38] C-reactive protein, immunoglobulins, interferons, interleukins, neutrophil products, other cytokines and chemokines are among the inflammatory signals that are associated with lung disease in CF.[38–43] A genome wide array search found that transforming growth factor β_1 is associated with early severe lung disease.[44] A case-control study found associations between mannose-binding lectin, TNF-α-238 genotype variations and poorer survival.[45] Our interest in HMGB-1[46,47] is due to proinflammatory effects associated with cell necrosis,[48] lung disease[49] and sepsis.[50] Recent reports note potential associations in CF.[51–54]

Biomarkers that predict subsequent key outcomes or survival seem the most likely candidates for being causal for progressive disease. In an observational study, sputum calprotectin levels were associated with shorter and longer times to next APE.[55] Our preliminary studies found two candidate biomarkers able to predict clinical outcomes in CF, HMGB-1[56] and free NE activity,[57] and additionally yielded one potential marker of APE severity, GM-CSF.[56]

Novel Predictive Biomarkers in CF

HMGB-1 plays a critical role shepherding DNA. It facilitates transcription by binding and modifying three dimensional DNA structure[58] and promotes DNA repair.[59] It has multiple other key roles that depend on an extensive array of post-translational modifications.[60–63] It was first described as a target for neurite outgrowth,[64] demonstrating a major role in development. The receptor for advanced glycation end-products (**RAGE**) is the key receptor for HMGB-1-mediated neurite growth.[65] However, RAGE is better known for its role in inflammation,[66–70] and many forms of HMGB-1 are pro-inflammatory signaling agents,[61–63] including the unmodified nuclear form that is released only after necrosis.[48] Released or secreted HMGB-1 mediates inflammatory arthritis,[71] sepsis,[50] acute lung injury,[49,72] post-traumatic injury associated coagulopathy and the systemic inflammatory response syndrome.[72] HMGB-1 blocking antibodies reverse arthritis[71] and produce remarkably improved, durable survival despite delayed

administration following septic shock induced by cecal ligation and puncture.[73] HMGB-1 was previously identified as associated with APE in CF.[52] In the CF airway, HMGB-1 attracts neutrophils,[52] prevents neutrophil efferocytosis[53] and amplifies the effects of bacterial lipopolysaccharide and cytosine-phosphatidyl-guanosine-DNA constructs.[74] Soluble RAGE, an antagonist to HMGB-1-RAGE binding, is absent in CF airways, potentiating HMGB-1 mediated inflammation.[51]

When released by neutrophil degranulation, NE can degrade matrix and damage tissue even in an environment replete with high affinity inhibitors due to transient micro-environmental effects.[75,76] Unopposed NE activity has long been implicated in pulmonary tissue damage.[77–79] Enzymatically active or free NE has been found in CF airways and is associated with airway pathology.[14,80–83] Free NE compromises opsonization, facilitates cellular binding of *Pseudomonas aeruginosa*[81,84] and promotes IL-8 release and production. NE is implicated in impairment of mucociliary clearance, abnormal airway remodeling and immunity impairments.[85]

GM-CSF maintains normal alveolar macrophage and innate immune responses to acute *P aeruginosa* pneumonia in mice.[86] In humans, COPD and asthma severity are associated with GM-CSF levels.[87] CF airway infections with *P aeruginosa* or *S aureus* elicit increased epithelial GM-CSF secretion[12] leading to prolonged survival and decreased apoptosis of airway neutrophils.[88] The resulting increase in airway neutrophils may prolong protease and reactive oxygen species releases.[10] These airway effects may be most prominent during an APE.

Specific Aims

This proposal has the **overall aim** is to discover or confirm specific sputum biomarkers that indicate disease state and predict key clinical events in CF. We plan rigorous patient selection, sample and data collection and data analysis to facilitate secure interpretation.[89] We propose to test **four specific hypotheses**: **(1)** HMGB-1 predicts time-to-first APE, **(2)** high HMGB-1 identifies patients likely to suffer an APE within 6 months, **(3)** NE predicts decline in FEV_1% over 2 years and **(4)** GMCSF measured at APE-onset is associated with the size of the accompanying FEV_1% decline from stable. To test these hypotheses and validate prior findings,[56,57] we propose **three specific aims**:

- **(1)** Assess the ability of HMGB-1 measured during a clinically stable state to predict time-to-first APE and identify patients most likely to suffer an APE within 6 months.
- **(2)** Assess the ability of NE to predict decline in FEV_1% during up to 2 years of follow up.
- **(3)** Assess the association of GMCSF measured at APE-onset with APE-associated FEV_1% decline.

We will evaluate HMGB-1 and NE as biomarkers that reflect clinical status and predict key outcomes in CF. We will test GM-CSF as an indicator of APE severity. Further, we will consider additional biomarkers as potential predictors of CF outcomes. Validation may identify mechanisms of disease and novel targets for treatment of airway inflammation. Discovery of additional predictive biomarkers will generate new testable hypotheses for mechanisms of disease. The biomarkers may provide new trial endpoints and clinical monitoring tools to improve strategies for preventing APE, pulmonary deterioration and early demise. This proposal answers the call for validated sputum biomarkers that predict disease progression, indicate APE onset and improve outcome measurements.[30]

Patient Selection and Enrollment

Adult patients may be included if they are able to give consent and have a confirmed diagnosis of CF and are able to expectorate sputum during a clinic visit. In the opinion of the enrolling investigator, the patient must be clinically stable at the time of enrollment.

Adult patients will be excluded if consent cannot be obtained, if they cannot produce sputum or they refuse to participate or if they have previously undergone solid organ or bone marrow transplantation or are on immunosuppressive medications beyond oral prednisone (such as methotrexate) or are on Xolair or other immunoglobulin-based medications. A history of a blood or platelet transfusion is not exclusionary. Prisoners, pregnant women and other vulnerable patients are excluded.

Adolescent patients, aged 12 to 17, may be included if they are able to give assent with a consenting guardian or parent and have a confirmed diagnosis of CF and have a history of producing sputum even if they have not been able to expectorate a sputum sample for collection, and they are willing to undergo sputum induction during a clinic visit. In the opinion of the enrolling investigator, the patient must be clinically stable at the time of enrollment.

Adolescent patients will be excluded if assent and permission cannot be obtained, if they cannot produce sputum or cannot undergo sputum induction or they refuse to participate or if they have previously undergone solid organ or bone marrow transplantation or are on immunosuppressive medications beyond oral prednisone (such as methotrexate) or are on Xolair or other immunoglobulin-based medications. A history of a blood or platelet transfusion is not exclusionary. Prisoners, pregnant women and other vulnerable patients are excluded.

Patients that become prisoners, pregnant or some other vulnerable status after enrollment do not need to be withdrawn from the study because the study is observational and there is no potential for coercive enrollment.

Any patient may be withdrawn at any time from the study by the patient, legal guardian or the investigator. Reporting the reason for withdrawal would be desirable but would not be required.

At the beginning of the study, each participating center will create a list of patients in alphabetical order by last name then first name then middle name as needed that would qualify for inclusion. The Utah center will generate a list of the same length of randomly chosen **Eligibility Letters** from A to Y for each center. More than one potential patient at a center may share the same Eligibility Letter. Each center will match their list of patients with the list of Eligibility Letters. It is absolutely essential for the sake of successful randomization that the order of patients and the order of Eligibility Letters be unchanged. Successful randomization greatly improves our chance of producing generalizable and believable results.

Each center will be assigned a **Selection Letterer** from B to Z.

Patients that fulfill inclusion and exclusion criteria will be approached for enrollment if and only if the **Eligibility Letter** for that patient is **earlier in the alphabet than** the **Selection Letter** for

that center.

Example 1: a center receives the Selection Letter of M. A patient receives the Eligibility Letter of B. This patient has a track record in the center of being exceptionally difficult to work with and often misses clinic appointments. This patient is a potential subject, and the Eligibility Letter of B may NOT be changed. A second patient receives the Eligibility Letter X. The second patient is exceptionally good about coming to clinic and participating in studies. This patient is not eligible because X follows M in the alphabet. This patient should not be approached for inclusion, and the Eligibility Letter of X may NOT be changed.

Example 2: Two patients with the same Eligibility Letter of F appear in clinic. The Selection Letter for the clinic is M. Both patients should be approached for enrollment if they both fulfil inclusion and exclusion criteria. Both patients agree to participate. Unique Study ID Numbers are obtained from REDCap for the patients, and the respective numbers are used on all specimen containers for the patients.

Example 3: a patient with an Eligibility Letter of F comes to clinic, and he or she fulfills all inclusion and exclusion criteria, and the site has a Selection Letter of K. This patient should be approached for enrollment.

Example 4: a patient without an Eligibility Letter comes to clinic and fulfills all inclusion and exclusion criteria. This patient should NOT be approached for enrollment.

Example 5: a patient with an Eligibility Letter of K comes to clinic, and the site has a Selection Letter of K; this patient should NOT be approached for enrollment.

Example 6: the day after a patient is not enrolled because his or her Eligibility Letter of M was later in the alphabet than the center's Selection Letter of L, the Interim Enrollment analysis is done and the Selection Letter is changed to N. DO NOT call the patient back for enrollment. That patient should be approached at the next clinic visit.

Study Design and Procedures

Randomization
The generation of **Eligibility Letters** includes randomization of patients for potential inclusion in this observational study prior to clinic. At the beginning of the study, each site will create a list of potential participants in alphabetical order by last name, then first name, then middle name. The Utah center will provide a list of random Eligibility Letters of equal length that will be matched to the list of potential participants by each site. None of the names on the list may be moved and none of the Eligibility Letters may be exchanged prior to matching the lists of names and Eligibility Letters. **Each site must maintain their list of patients and matched Eligibility Letters for the duration of the study to allow data monitoring but also maintain patient confidentiality for patients that are not enrolled.**

Illustration: Monitoring will occur during the study. The Utah center will visit sites and include a check of the success of randomization. The Utah center will generate a list of enrolled patients and their respective Eligibility Letters for each site based on data submitted via the REDCap system. Site personnel will be asked to verify the Utah list against the local site list of patients and Eligibility Letters.

Selection Letters and enrollment will be reviewed after the first month and then quarterly. At these times Selection Letters may be changed to adjust each site's enrollment rate as needed.

Study ID Number
The Study ID Number will be the number associated with the enrolled patient and will be obtained through REDCap. Click the link to "Add/Edit Records", then "Add new record". A unique Study ID will be assigned to this patient. This Study ID Number is the number that should be used to label all samples and study data.

Duration of Follow Up
All patients will be followed for a minimum of one year after enrollment. The first patient enrolled in the overall study will be followed for up to two years. The study observation period ends one year after enrollment of the last patient in the study or two years after the enrollment of the first patient, whichever comes later.

Sputum Collections

First sputum collection
After written informed consent, stable patients, as determined by the investigator, will provide samples within 48 hours of a clinic visit. Ideally, the study sample will be collected in a tube concurrently with any clinical sample. Adult patients will provide sputum via expectoration. Adolescent patients may undergo sputum induction if this is an existing option for standard clinical practice. Patients will be given two tubes and will cough into each one, alternating tubes with each cough. If insufficient sputum is collected for study purposes, it is permissible for patients to return to clinic within 48 hours to provide a sample. Samples should be labeled Sample 1 with the patient's unique Study ID Number and date of collection.

For the first sputum collection, sputum induction is not allowed for adults because the primary endpoints of the study depend on measurements of HMGB-1 from expectorated sputum. There is a technical issue in that HMGB-1 may not be measurable under high salt conditions which would cripple our ability to complete the primary aim of the study. Accordingly, patients should not be given hypertonic saline as a treatment during clinic before sputum collection. Hypertonic saline treatment at home prior to coming to clinic is not a problem.

If a patient that appears eligible for enrollment no-shows or cancels a clinic visit, the study coordinator may make one contact with the patient to encourage rescheduling the visit. Patients must be seen in clinic to be enrolled in the study.

Second sputum collection

Immediately after diagnosis of the first acute pulmonary exacerbation (defined below, **APE**) following enrollment, a sputum will be collected from each enrolled patient. There is a 48 hour window for collection to allow for communication delays if a patient is hospitalized from an Emergency Department after hours or during a weekend. However, this means that Friday evening admissions will need to be collected by Sunday evening; waiting until Monday (72 hours) may be too long, and the effects of antibiotic or other treatments may cloud the interpretation of our biomarker measurement results. We prefer sample collection as close to the time of diagnosis as possible, most preferably within an hour or two. For adolescent and adult patients that cannot produce expectorated sputum, sputum induction is allowed provided that this is an existing option for standard clinical practice. Samples should be labeled Sample 2 with the patient's unique Study ID Number and date of collection. If no sample can be collected for some reason when a patient suffers an APE, we still need the data collection form filled out. We anticipate that some patients will not have an APE at all by the end of the trial, thus no APE sample may be collected.

Third Sputum Sample

Eight weeks ± 4 weeks after APE onset, at a clinic follow up visit, a sputum sample should be collected and the patient's clinical status noted as stable, mild APE or APE. For adolescent and adult patients that cannot produce expectorated sputum, sputum induction is allowed provided that this is an existing option for standard clinical practice. Samples should be labeled Sample 3 with the patient's unique Study ID Number and date of collection. If a sample cannot be collected, the data collection form should still be completed.

Fourth Sputum Sample

A fourth and final sample should be collected at the next regular appointment after sample 3 or at the next APE, whichever comes first. For adolescent and adults that cannot produce expectorated sputum, sputum induction is allowed provided that this is an existing option for standard clinical practice. Samples should be labeled Sample 4 with the patient's unique Study ID Number and date of collection. If a sputum sample cannot be collected, the data collection form should still be completed.

Patients without APE following enrollment

As mentioned above, some patients will not have an APE during the year after enrollment. For these patients, a second and final sample should be obtained around the 1 year point following

enrollment. Samples should be labeled Sample 4 with the patient's unique Study ID Number and date of collection. For adolescent and adult patients that cannot produce expectorated sputum, sputum induction is allowed. For these patients, follow up should continue until the end of the study.
If one of these patients has an APE during the second year of follow up, a sputum sample should be collected and treated like a Second Sputum Sample. However, to avoid confusion, the sample should be labeled Sample 5 with the patient's unique Study ID Number and date of collection.

Patients with respiratory arrest or failure with intubation and mechanical ventilation
We hope that few if any enrolled patients will present with an APE and need intubation and mechanical ventilation. Sputum samples may still be collected from these patients via tracheal aspirate within 48 hours of APE diagnosis. These samples should be labeled Sample 2, 3, 4 or 5 as appropriate with the patient's unique Study ID Number and date of collection.

Patients with respiratory failure and non-invasive mechanical ventilation
We equally hope that few if any enrolled patients will present with an APE in respiratory failure require non-invasive mechanical ventilation. Sputum collection in these patients may be difficult or increase the risk of requirement for intubation and mechanical ventilation. The investigator should exercise caution and discretion in obtaining any sputum samples. These samples should be labeled Sample 2, 3, 4 or 5 as appropriate with the patient's unique Study ID Number and date of collection.

Sputum samples should be processed according to the Sputum Processing Instructions. Samples will be frozen after processing at each site and batch-shipped. A small amount of processed sputum will be retained by each site as a physical back up collection of sputum.

<u>**Definitions**</u>

Clinically Stable:

- No increase in symptoms
- No objective findings of an APE (see below)
- At PI discretion for borderline cases

Acute Pulmonary Exacerbation[90] (**APE**):

- One or more subjective acute symptoms (in addition to chronic symptoms or above patient's baseline)
    - Increased sputum, cough, dyspnea
    - Chest pain or tightness
    - Hemoptysis, fever, chills, arthralgias, fatigue
- One or more objective acute findings
    - 10% drop in FEV_1 or FVC
    - Temp > 38.4 C
    - Witness hemoptysis greater than 100 mL per episode
    - SaO_2 < 90% or PaO_2 < 60 mm Hg despite usual oxygen
    - For adolescents a drop in SaO_2 of 5% (for example, 97% to 92%)
    - Increased supplemental oxygen
    - Unplanned weight loss ≥ 5% of baseline body weight over 3 months

Respiratory arrest or failure requiring mechanical ventilation regardless of other criteria
At PI discretion for borderline cases or for cases with serious findings not included here

If a patient is not stable, but does not fulfill criteria for an APE, classify the patient as **Mild APE**. These patients will either lack symptoms or lack objective findings. Investigators will report Mild APE. Potential study patients with Mild APE should not be enrolled. If Mild APE is present at the first post-APE visit, Sputum Sample 3 should still be collected. This sample should be labeled Sample 3 with the patient's unique Study ID Number and date of collection.

Scenarios for Sputum Collections associated with an APE

Example 7: A patient complains of increased shortness of breath and new chest tightness, but no objective findings are present. This patient is classified as having a mild APE. The investigator or research coordinator should not enroll this patient in the study, but the patient is eligible for enrollment at a future clinic visit if he or she is stable at that time.

Example 8: A patient already enrolled in the study complains of increased shortness of breath and new chest tightness, and has a temperature of 38.2. This is the first visit since enrollment. The patient is classified as having a mild APE. The investigator or research coordinator completes an APE reporting form but does not collect a sputum sample.

Example 9: An enrolled patient complains of increased shortness of breath and new chest tightness, and has a temperature of 38.2. This patient is classified as having a mild APE using strict criteria. However the physician feels that the patient appears toxic and finds a blood pressure that is somewhat low compared to that patient's usual BP. Because of the investigator's option concerning other serious findings, the investigator decides to classify this as an APE. The investigator or research coordinator completes an APE reporting form. If this is the first APE following enrollment, Sputum Sample 2 is collected and the sputum collection form is completed.

Example 10: An enrolled patient has an SaO_2 of 88% but denies all symptoms. This patient is classified as having a mild APE. The investigator or research coordinator completes an APE reporting form.

Example 11: An enrolled patient appears at the Emergency Department for a participating institution and has respiratory arrest, undergoes intubation and begins mechanical ventilation. This patient is classified as having an APE. The investigator or research coordinator completes an APE reporting form. Tracheal aspiration of sputum is performed as part of ventilator associated care. Sputum from tracheal aspiration is collected and submitted along with a sputum collection form.

Example 12: An enrolled patient appears at the Emergency Department for a participating institution and has near-respiratory arrest and begins non-invasive mechanical ventilation via bi-level positive airway pressure (BiPAP). The patient is unable to tolerate any time off BiPAP. This patient is classified as having an APE. The investigator or research coordinator completes an APE reporting form. Sputum collection is not performed unless the primary purpose of sputum production is airway clearance for patient care and NOT the study. In close cooperation

with the patient's care team, a sputum sample may be collected within 48 hours of admission as a byproduct of airway clearance for clinical care. If a sample is collected, a sputum collection form is completed.

In case of doubt, consultation with Drs. Liou or Sagel is requested, but decisions regarding stable state or APE state are to be made by the investigator seeing the patient at the time of clinic visit.

Sample Analysis

We will assay HMGB-1 with commercially available antibodies (R&D Systems Inc., Minneapolis, MN) and published ELISA methods in Utah.[91] GMCSF, GCSF, IFN-α, IL-1β, IL-5, IL-8, IL-17 and potentially other candidate biomarkers will be assayed by multiplex ELISA (Searchlight, Aushon, Billerica, MA). We will transport aliquots of sputum fractions for all patients to the Center for Biochemical Markers at Children's Hospital Colorado to provide an off-site duplicate sample repository protected by redundant power and alarms. NE, proteinase-3 and cathepsin-G activities and protease inhibitory activities will be assessed in Colorado by active site titration with spectrofluorometric measurements using proteinase specific ortho-aminobenzoyl-peptidyl-*N*-(2,4-dintrophenyl) ethylene-diamine fluorescence resonance energy transfer substrates[92] similar to methods we used previously.[57,76,93] Calprotectin will be assayed by stand-alone ELISA in Colorado.[55] Transport of frozen aliquoted samples to Colorado or Aushon will be done by courier to avoid disastrous losses.[56]

Data Collection

We will record demographics, CF Registry number, smoking status, lung function performed in accordance with ATS standards,[94] prior-year APE and hospitalizations, questions regarding sleep, nutrition, dyspnea, pain, pregnancy, date of last menstruation, vital signs, historic and current microbiology including bacterial, atypical mycobacterial, viral, and fungal results and current treatments (especially anti-inflammatory), simultaneously with sputum collections. Participation in any other trial at any time during our study will be noted along with the nature of the trial (observational or interventional). Data will be collected at clinic visits, APE admissions, follow-up clinic visits, death or lung transplant listing and actual transplant and at the end of the study. The new data will have the quality and statistical power to give strong confirmation of the previous findings and, we hope, to give totally new insights.

We will collect, manage and store data in the Biomedical Research Informatics Service Core (BRISC) using the Research Electronic Data Capture (REDCap) system.[95] REDCap enables in-line validation to minimize transcription errors and provides real-time notifications of data submission thus allowing immediate central monitoring and feedback. The system is compliant with 21 CFR 11 and 45 CFR 46 regarding protection of human subjects and data management. Data are stored within the U of Utah Center for High Performance Computing virtual machine protected space. Access to data requires an encrypted secure socket layer (SSL) connection, a standard capability of all modern web browsers. Data access (project, data collection instrument) creation, edits and other changes are logged by user ID, time stamp and project. These logs are permanently retained by the REDCap system. Databases are backed up every 2 hours, nightly, weekly and monthly with each level encompassed by the next. Monthly backups are removed to off site storage as part of the BRISC disaster recovery plan. A portable document format sample REDCap data entry eCRF is included in the Appendix. We plan to review newly collected data on a daily basis and make corrections immediately in order to

maximize data accuracy.

Statistical Analysis Plan

Data Security to Enable Reliable Interpretation[89]

Following study initiation at each MWCFC site, we plan interim analyses within 6 months to identify and correct center specific biases in particular. We will assay up to the first 10 samples per participating site. Assay results for patients within each center will be evaluated by summary statistics and ANOVA to identify centers with high variation between similar stable patients. Linear regression of FEV_1% by each biomarker result will be performed including a categorical variable denoting each CF center. Center-specific bias will be identified if the center category variable is statistically significant.

At the end of sputum and data collection, HMGB-1 ELISA, multiplex ELISA assays and protease activity assays will each be performed in a single batch to eliminate batch-to-batch variations.

Main statistical analyses

Specific Aim 1: *Assess the ability of HMGB-1 measured during a clinically stable state to predict time-to-first APE and identify patients most likely to suffer an APE within 6 months.*

Testing hypothesis 1 (first part): *HMGB-1 predicts time-to-first APE*. We will perform univariate proportional hazards modeling[96,97] of time-to-first APE with each biomarker measured in the enrollment, clinically stable-state sputum samples. All biomarkers with a p-value < 0.2 will be included in multivariate modeling with backward selection procedures. We will evaluate successive interim models by considering maximum log likelihood. The final interim candidate model will be subjected to forward selection procedures to reduce the possibility that order of elimination caused mistaken removal of a biomarker and to evaluate any associations with clinical covariates such as age, gender, lung function, airway infections, anti-inflammatory treatments, collection season, MWCFC site or other clinical parameter at the time of sputum collection. Inclusion of other potential biomarkers, cytokines or proteinase activities, with backwards selection will allow us to test whether HMGB-1 alone is sufficient to predict time-to-first APE as we found in our preliminary data. We are particularly interested in the possibility that calprotectin will remain in the model after backward selection due to the similarity with HMGB-1 of predictive ability for time-to-first APE.[55]

Testing hypothesis 2: *high HMGB-1 identifies patients likely to suffer an APE within 6 months*. Using subsequent diagnosis of an APE within 6 months as the clinical discriminator, we will perform receiver-operator characteristic (ROC) evaluation[98] of each biomarker and selected clinical covariates. For each ROC curve generated, we will calculate the area under the curve (AUC or accuracy) and its 95% confidence interval by sampling covariate data with replacement and bootstrapping with 1000 iterations.[56,99]

Specific Aim 2: *Assess the ability of NE to predict decline in FEV_1% during up to 2 years of follow up.*

Testing hypothesis 3: *NE predicts decline in FEV_1% over 2 years*. We will calculate the actual fall in FEV_1%, and the rate of decline using linear regression and linear mixed effects regression.[10,100] Using these values as the outcomes, we will perform univariate linear regression modeling with NE and the other biomarkers. We will perform multivariate linear regression starting with all biomarkers with $p < 0.2$ and apply backward selection procedures, evaluating

successive models with maximum log likelihoods. Eliminated covariates and clinical covariates will assessed by forward selection methods as above.

Specific Aim 3: *Assess the association of GMCSF measured at APE-onset with APE-associated FEV_1% drop.*

Testing hypothesis 4: *GMCSF measured at APE-onset is associated with APE-associated declines in FEV_1% from stable clinical states.* Recruitment projections suggest that we are likely to enroll about 100 patients that have an APE within one year. Within 2 years, it is likely that the number of APE among unique individuals will meet or exceed the sample size calculated above. For patients with APE, we will calculate the acute declines in FEV_1% compared to the values obtained at study enrollment and the most recent stable-state clinic visit. We will examine biomarker measurements obtained at enrollment in the stable state, obtained simultaneously with the APE and changes in biomarker measurements between stable and APE states. We will use univariate linear regression to evaluate associations between acute decline in FEV_1% and biomarkers. We previously found that IL-5 was a significant independent predictor of FEV_1% decline. Thus, we will perform multivariate analysis as described in the two prior specific aims with backwards and forward selection procedures to see if the relationships between GM-CSF and IL-5 with FEV_1% decline persist. Modeling will demonstrate whether other biomarkers are contributory.

Accomplishing our specific aims will validate our prior discoveries or add new biomarkers to the list of predictive and therefore potentially causal agents for inflammation. The size of the trial combined with the effort to reduce biases and improve interpretability suggests that we have the potential to make new biomarker discoveries. Validation of our discoveries will confirm new insights into the pathophysiology and direct mechanistic investigations of airway inflammation. Validated biomarkers will be of great use in providing surrogate markers of therapeutic efficacy for new potential agents. Confirmation of one or more biomarkers that report on severity of an APE potentially provides a new tool for assessing and optimizing acute APE therapies. The careful clinical annotation of the collected samples will allow future investigations with high likelihood of reliable interpretation thus promising additional high value results beyond those specifically sought in this proposal.

Data Monitoring

This is an observational study and represents minimal risk to subjects. A Data Safety Monitoring Board is not required.

On site data monitoring and source document verification will be performed. Sputum processing techniques will be observed for accuracy in following the processing instructions. Veracity of sample labeling will be confirmed.

An advisory committee has been established composed of three distinguished outside scientists familiar with lung disease, biomarker research, statistical methods and cystic fibrosis.

References

1. Andersen DH. Cystic fibrosis of the pancreas and its relation to celiac disease: A clinical and pathologic study. *Am J Dis Child*. 1938;56(2):344–399.

doi:10.1001/archpedi.1938.01980140114013.

2. Shwachman H, Kulczycki LL. Long-term study of one hundred five patients with cystic fibrosis; studies made over a five- to fourteen-year period. *Ama J. Dis. Child.* 1958;96(1):6–15.

3. Davis PB. Cystic Fibrosis Since 1938. *Am J Respir Crit Care Med*. 2006;173(5):475–482. doi:10.1164/rccm.200505-840OE.

4. Gibson RL, Burns JL, Ramsey BW. Pathophysiology and management of pulmonary infections in cystic fibrosis. *Am. J. Respir. Crit. Care Med.* 2003;168(8):918–51. doi:10.1164/rccm.200304-505SO.

5. Taussig LM, Kattwinkel J, Friedewald WT, Di Sant'Agnese PA. A new prognostic score and clinical evaluation system for cystic fibrosis. *J. Pediatr.* 1973;82(3):380–390.

6. Kerem E, Reisman J, Corey M, Canny GJ, Levison H. Prediction of mortality in patients with cystic fibrosis. *N. Engl. J. Med.* 1992;326(18):1187–1191.

7. Hayllar KM, Williams SG, Wise AE, et al. A prognostic model for the prediction of survival in cystic fibrosis. *Thorax*. 1997;52(4):313–317.

8. Corey M, Farewell V. Determinants of mortality from cystic fibrosis in Canada, 1970-1989. *Am. J. Epidemiol.* 1996;143(10):1007–1017.

9. Aurora P, Wade A, Whitmore P, Whitehead B. A model for predicting life expectancy of children with cystic fibrosis. *Eur. Respir. J. Off. J. Eur. Soc. Clin. Respir. Physiol.* 2000;16(6):1056–1060.

10. Liou TG, Adler FR, Fitzsimmons SC, Cahill BC, Hibbs JR, Marshall BC. Predictive 5-year survivorship model of cystic fibrosis. *Am. J. Epidemiol.* 2001;153(4):345–52. doi:10.1093/aje/153.4.345.

11. Buzzetti R, Alicandro G, Minicucci L, et al. Validation of a predictive survival model in Italian patients with cystic fibrosis. *J. Cyst. Fibros. Off. J. Eur. Cyst. Fibros. Soc.* 2012;11(1):24–29. doi:10.1016/j.jcf.2011.08.007.

12. *Cystic Fibrosis Foundation Patient Registry 2010 Annual Data Report*. Bethesda, Maryland: Cystic Fibrosis Foundation; 2011. Available at: http://www.cff.org/LivingWithCF/CareCenterNetwork/PatientRegistry/. Accessed June 21, 2012.

13. Division SS. Historical Statistics - The 2012 Statistical Abstract - U.S. Census Bureau, Expectation of Life at Birth by Race and Sex. Available at: http://www.census.gov/compendia/statab/hist_stats.html. Accessed August 26, 2012.

14. Konstan MW, Hilliard KA, Norvell TM, Berger M. Bronchoalveolar lavage findings in cystic fibrosis patients with stable, clinically mild lung disease suggest ongoing infection and inflammation. *Am. J. Respir. Crit. Care Med.* 1994;150(2):448–54.

15. Van Heeckeren AM, Schluchter MD, Xue W, Davis PB. Response to Acute Lung Infection with Mucoid Pseudomonas aeruginosa in Cystic Fibrosis Mice. *Am J Respir Crit Care Med*. 2006;173(3):288–296. doi:10.1164/rccm.200506-917OC.

16. Zemanick ET, Wagner BD, Harris JK, Wagener JS, Accurso FJ, Sagel SD. Pulmonary exacerbations in cystic fibrosis with negative bacterial cultures. *Pediatr. Pulmonol.* 2010;45(6):569–577. doi:10.1002/ppul.21221.

17. Konstan MW, Berger M. Current understanding of the inflammatory process in cystic fibrosis: onset and etiology. *Pediatr. Pulmonol.* 1997;24(2):137–42; discussion 159–61.

18. Fuchs HJ, Borowitz DS, Christiansen DH, et al. Effect of Aerosolized Recombinant Human DNase on Exacerbations of Respiratory Symptoms and on Pulmonary Function in Patients with Cystic Fibrosis. *N Engl J Med*. 1994;331(10):637–642. doi:10.1056/NEJM199409083311003.

19. Elkins MR, Robinson M, Rose BR, et al. A Controlled Trial of Long-Term Inhaled Hypertonic Saline in Patients with Cystic Fibrosis. *N Engl J Med*. 2006;354(3):229–240. doi:10.1056/NEJMoa043900.

20. Ramsey BW, Pepe MS, Quan JM, et al. Intermittent Administration of Inhaled Tobramycin in Patients with Cystic Fibrosis. *N Engl J Med*. 1999;340(1):23–30. doi:10.1056/NEJM199901073400104.

21. Retsch-Bogart GZ, Quittner AL, Gibson RL, et al. Efficacy and safety of inhaled aztreonam lysine for airway pseudomonas in cystic fibrosis. *Chest*. 2009;135(5):1223–1232. doi:10.1378/chest.08-1421.

22. Saiman L, Marshall BC, Mayer-Hamblett N, et al. Azithromycin in patients with cystic fibrosis chronically infected with Pseudomonas aeruginosa: a randomized controlled trial. *Jama J. Am. Med. Assoc.* 2003;290(13):1749–1756. doi:10.1001/jama.290.13.1749.

23. Cystic Fibrosis Foundation. *Cystic Fibrosis Foundation Patient Registry: 2008 annual data report to the center directors*. Bethesda, Maryland: Cystic Fibrosis Foundation; 2009.

24. Auerbach HS, Williams M, Kirkpatrick JA, Colten HR. Alternate-day prednisone reduces morbidity and improves pulmonary function in cystic fibrosis. *Lancet*. 1985;2(8457):686–688.

25. Regelmann WE, Elliott GR, Warwick WJ, Clawson CC. Reduction of sputum Pseudomonas aeruginosa density by antibiotics improves lung function in cystic fibrosis more than do bronchodilators and chest physiotherapy alone. *Am. Rev. Respir. Dis.* 1990;141(4 Pt 1):914–921.

26. McCoy KS, Quittner AL, Oermann CM, Gibson RL, Retsch-Bogart GZ, Montgomery AB. Inhaled aztreonam lysine for chronic airway Pseudomonas aeruginosa in cystic fibrosis. *Am. J. Respir. Crit. Care Med.* 2008;178(9):921–928. doi:10.1164/rccm.200712-1804OC.

27. Ramsey BW, Davies J, McElvaney NG, et al. A CFTR potentiator in patients with cystic fibrosis and the G551D mutation. *N. Engl. J. Med.* 2011;365(18):1663–1672. doi:10.1056/NEJMoa1105185.

28. Smith AL, Doershuk C, Goldmann D, et al. Comparison of a beta-lactam alone versus beta-lactam and an aminoglycoside for pulmonary exacerbation in cystic fibrosis. *J. Pediatr.* 1999;134(4):413–421.

29. Saiman L, Anstead M, Mayer-Hamblett N, et al. Effect of azithromycin on pulmonary function in patients with cystic fibrosis uninfected with Pseudomonas aeruginosa: A randomized controlled trial. *JAMA*. 2010;303(17):1707–1715. doi:10.1001/jama.2010.563.

30. Sagel SD, Chmiel JF, Konstan MW. Sputum biomarkers of inflammation in cystic fibrosis lung disease. *Proc Am Thorac Soc*. 2007;4(4):406–417. doi:10.1513/pats.200703-044BR.

31. Bonfield TL, Panuska JR, Konstan MW, et al. Inflammatory cytokines in cystic fibrosis lungs. *Am. J. Respir. Crit. Care Med.* 1995;152(6 Pt 1):2111–8.

32. Fidler KJ, Hilliard TN, Bush A, et al. Mannose-binding lectin is present in the infected airway: a possible pulmonary defence mechanism. *Thorax*. 2009;64(2):150–155. doi:10.1136/thx.2008.100073.

33. Hubeau C, Le Naour R, Abély M, et al. Dysregulation of IL-2 and IL-8 production in circulating T lymphocytes from young cystic fibrosis patients. *Clin. Exp. Immunol.* 2004;135(3):528–534.

34. Koller DY, Nething I, Otto J, Urbanek R, Eichler I. Cytokine concentrations in sputum from patients with cystic fibrosis and their relation to eosinophil activity. *Am. J. Respir. Crit. Care Med.* 1997;155(3):1050–1054.

35. Frederiksen B, Koch C, Høiby N. Antibiotic treatment of initial colonization with Pseudomonas aeruginosa postpones chronic infection and prevents deterioration of pulmonary function in cystic fibrosis. *Pediatr. Pulmonol.* 1997;23(5):330–335. doi:10.1002/(SICI)1099-0496(199705)23:5<330::AID-PPUL4>3.0.CO;2-O.

36. Kosorok MR, Zeng L, West SE, et al. Acceleration of lung disease in children with cystic fibrosis after Pseudomonas aeruginosa acquisition. *Pediatr. Pulmonol.* 2001;32(4):277–287.

37. Dasenbrook EC, Merlo CA, Diener-West M, Lechtzin N, Boyle MP. Persistent methicillin-resistant Staphylococcus aureus and rate of FEV1 decline in cystic fibrosis. *Am. J. Respir. Crit. Care Med.* 2008;178(8):814–821. doi:10.1164/rccm.200802-327OC.

38. Mayer-Hamblett N, Aitken ML, Accurso FJ, et al. Association between pulmonary function and sputum biomarkers in cystic fibrosis. *Am J Respir Crit Care Med*. 2007;175(8):822–828. doi:10.1164/rccm.200609-1354OC.

39. Sagel SD, Sontag MK, Wagener JS, Kapsner RK, Osberg I, Accurso FJ. Induced sputum inflammatory measures correlate with lung function in children with cystic fibrosis. *J. Pediatr.* 2002;141(6):811–7.

40. Sagel SD, Kapsner RK, Osberg I. Induced sputum matrix metalloproteinase-9 correlates with lung function and airway inflammation in children with cystic fibrosis. *Pediatr. Pulmonol.*

2005;39(3):224–32. doi:10.1002/ppul.20165.

41. Hartl D, Griese M, Kappler M, et al. Pulmonary T(H)2 response in Pseudomonas aeruginosa-infected patients with cystic fibrosis. *J. Allergy Clin. Immunol.* 2006;117(1):204–211. doi:10.1016/j.jaci.2005.09.023.

42. Kim J-S, Okamoto K, Rubin BK. Pulmonary function is negatively correlated with sputum inflammatory markers and cough clearability in subjects with cystic fibrosis but not those with chronic bronchitis*. *Chest*. 2006;129(5):1148–1154. doi:10.1378/chest.129.5.1148.

43. Tan H-L, Regamey N, Brown S, Bush A, Lloyd CM, Davies JC. The Th17 Pathway in Cystic Fibrosis Lung Disease. *Am. J. Respir. Crit. Care Med.* 2011. doi:10.1164/rccm.201102-0236OC.

44. Drumm ML, Konstan MW, Schluchter MD, et al. Genetic modifiers of lung disease in cystic fibrosis. *N Engl J Med*. 2005;353(14):1443–1453. doi:10.1056/NEJMoa051469.

45. Yarden J, Radojkovic D, De Boeck K, et al. Polymorphisms in the mannose binding lectin gene affect the cystic fibrosis pulmonary phenotype. *J. Med. Genet.* 2004;41(8):629 –633. doi:10.1136/jmg.2003.017947.

46. Liou TG. Quantitative relationships between markers of inflammation and clinical disease in cystic fibrosis. *Pediatr. Pulmonol.* 2006;41(S29):254. doi:10.1002/ppul.20538.

47. Liou TG. The correlation of high mobility group box-1 protein, a pro-inflammatory biomarker, with clinical outcomes in CF. *Pediatr. Pulmonol.* 2009;44(S32):236. doi:10.1002/ppul.21133.

48. Scaffidi P, Misteli T, Bianchi ME. Release of chromatin protein HMGB1 by necrotic cells triggers inflammation. *Nature*. 2002;418(6894):191–195. doi:10.1038/nature00858.

49. Ueno H, Matsuda T, Hashimoto S, et al. Contributions of high mobility group box protein in experimental and clinical acute lung injury. *Am. J. Respir. Crit. Care Med.* 2004;170(12):1310–1316. doi:10.1164/rccm.200402-188OC.

50. Yang H, Ochani M, Li J, et al. Reversing established sepsis with antagonists of endogenous high-mobility group box 1. *Proc. Natl. Acad. Sci. U. S. A.* 2004;101(1):296–301. doi:10.1073/pnas.2434651100.

51. Makam M, Diaz D, Laval J, et al. Activation of critical, host-induced, metabolic and stress pathways marks neutrophil entry into cystic fibrosis lungs. *Proc. Natl. Acad. Sci. U. S. A.* 2009;106(14):5779–5783. doi:10.1073/pnas.0813410106.

52. Rowe SM, Jackson PL, Liu G, et al. Potential role of high-mobility group box 1 in cystic fibrosis airway disease. *Am. J. Respir. Crit. Care Med.* 2008;178(8):822–831. doi:10.1164/rccm.200712-1894OC.

53. Liu G, Wang J, Park Y-J, et al. High mobility group protein-1 inhibits phagocytosis of apoptotic neutrophils through binding to phosphatidylserine. *J. Immunol. Baltim. Md 1950*.

2008;181(6):4240–4246.

54. Gaggar A, Rowe SM, Matthew H, Blalock JE. Proline-Glycine-Proline (PGP) and High Mobility Group Box Protein-1 (HMGB1): Potential Mediators of Cystic Fibrosis Airway Inflammation. *Open Respir Med J*. 4:32–38. doi:10.2174/1874306401004020032.

55. Gray RD, Imrie M, Boyd AC, Porteous D, Innes JA, Greening AP. Sputum and serum calprotectin are useful biomarkers during CF exacerbation. *J. Cyst. Fibros. Off. J. Eur. Cyst. Fibros. Soc.* 2010;9(3):193–198. doi:10.1016/j.jcf.2010.01.005.

56. Liou TG, Adler FR, Keogh RH, et al. Sputum Biomarkers and the Prediction of Clinical Outcomes in Patients with Cystic Fibrosis. Kreindler JL, ed. *Plos One*. 2012;7(8):e42748. doi:10.1371/journal.pone.0042748.

57. Sagel SD, Wagner BD, Anthony MM, Emmett P, Zemanick ET. Sputum Biomarkers of Inflammation and Lung Function Decline in Children with Cystic Fibrosis. *Am. J. Respir. Crit. Care Med.* 2012. doi:10.1164/rccm.201203-0507OC.

58. Maher JF, Nathans D. Multivalent DNA-binding properties of the HMG-1 proteins. *Proc. Natl. Acad. Sci. U. S. A.* 1996;93(13):6716–6720.

59. Lange SS, Mitchell DL, Vasquez KM. High mobility group protein B1 enhances DNA repair and chromatin modification after DNA damage. *Proc. Natl. Acad. Sci.* 2008;105(30):10320–10325. doi:10.1073/pnas.0803181105.

60. Chao YB, Scovell WM, Yan SB. High mobility group protein, HMG-1, contains insignificant glycosyl modification. *Protein Sci. Publ. Protein Soc.* 1994;3(12):2452–2454. doi:10.1002/pro.5560031230.

61. Bonaldi T, Talamo F, Scaffidi P, et al. Monocytic cells hyperacetylate chromatin protein HMGB1 to redirect it towards secretion. *Embo J.* 2003;22(20):5551–5560. doi:10.1093/emboj/cdg516.

62. Ito I, Fukazawa J, Yoshida M. Post-translational methylation of high mobility group box 1 (HMGB1) causes its cytoplasmic localization in neutrophils. *J. Biol. Chem.* 2007;282(22):16336–16344. doi:10.1074/jbc.M608467200.

63. Venereau E, Casalgrandi M, Schiraldi M, et al. Mutually exclusive redox forms of HMGB1 promote cell recruitment or proinflammatory cytokine release. *J. Exp. Med.* 2012;209(9):1519–1528. doi:10.1084/jem.20120189.

64. Merenmies J, Pihlaskari R, Laitinen J, Wartiovaara J, Rauvala H. 30-kDa heparin-binding protein of brain (amphoterin) involved in neurite outgrowth. Amino acid sequence and localization in the filopodia of the advancing plasma membrane. *J. Biol. Chem.* 1991;266(25):16722–9.

65. Hori O, Brett J, Slattery T, et al. The receptor for advanced glycation end products (RAGE) is a cellular binding site for amphoterin. Mediation of neurite outgrowth and co-expression of rage

and amphoterin in the developing nervous system. *J. Biol. Chem.* 1995;270(43):25752–25761.

66. Schmidt AM, Yan SD, Yan SF, Stern DM. The biology of the receptor for advanced glycation end products and its ligands. *Biochim. Biophys. Acta*. 2000;1498(2-3):99–111.

67. Liliensiek B, Weigand MA, Bierhaus A, et al. Receptor for advanced glycation end products (RAGE) regulates sepsis but not the adaptive immune response. *J. Clin. Invest.* 2004;113(11):1641–1650. doi:10.1172/JCI18704.

68. Herold K, Moser B, Chen Y, et al. Receptor for advanced glycation end products (RAGE) in a dash to the rescue: inflammatory signals gone awry in the primal response to stress. *J. Leukoc. Biol.* 2007;82(2):204–212. doi:10.1189/jlb.1206751.

69. Reynolds PR, Schmitt RE, Kasteler SD, et al. Receptors for advanced glycation end-products targeting protect against hyperoxia-induced lung injury in mice. *Am. J. Respir. Cell Mol. Biol.* 2010;42(5):545–551. doi:10.1165/rcmb.2008-0265OC.

70. Reynolds PR, Wasley KM, Allison CH. Diesel particulate matter induces receptor for advanced glycation end-products (RAGE) expression in pulmonary epithelial cells, and RAGE signaling influences NF-κB-mediated inflammation. *Environ. Health Perspect.* 2011;119(3):332–336. doi:10.1289/ehp.1002520.

71. Kokkola R, Li J, Sundberg E, et al. Successful treatment of collagen-induced arthritis in mice and rats by targeting extracellular high mobility group box chromosomal protein 1 activity. *Arthritis Rheum.* 2003;48(7):2052–2058. doi:10.1002/art.11161.

72. Cohen MJ, Brohi K, Calfee CS, et al. Early release of high mobility group box nuclear protein 1 after severe trauma in humans: role of injury severity and tissue hypoperfusion. *Crit. Care Lond. Engl.* 2009;13(6):R174. doi:10.1186/cc8152.

73. Wang H, Bloom O, Zhang M, et al. HMG-1 as a late mediator of endotoxin lethality in mice. *Science*. 1999;285(5425):248–251. doi:10.1126/science.285.5425.248.

74. Hreggvidsdottir HS, Ostberg T, Wähämaa H, et al. The alarmin HMGB1 acts in synergy with endogenous and exogenous danger signals to promote inflammation. *J. Leukoc. Biol.* 2009;86(3):655–662. doi:10.1189/jlb.0908548.

75. Liou TG, Campbell EJ. Nonisotropic enzyme--inhibitor interactions: a novel nonoxidative mechanism for quantum proteolysis by human neutrophils. *Biochemistry (Mosc.)*. 1995;34(49):16171–7.

76. Liou TG, Campbell EJ. Quantum proteolysis resulting from release of single granules by human neutrophils: a novel, nonoxidative mechanism of extracellular proteolytic activity. *J. Immunol. Baltim. Md 1950*. 1996;157(6):2624–31.

77. Carp H, Miller F, Hoidal JR, Janoff A. Potential mechanism of emphysema: alpha 1-proteinase inhibitor recovered from lungs of cigarette smokers contains oxidized methionine and has decreased elastase inhibitory capacity. *Proc. Natl. Acad. Sci. U. S. A.* 1982;79(6):2041–5.

78. Hoidal JR, Niewoehner DE, Rao NV, Hibbs MS. The role of neutrophils in the development of cadmium chloride-induced emphysema in lathyrogen-fed hamsters. *Am. J. Pathol.* 1985;120(1):22–9.

79. Hoidal JR, Niewoehner DE. Pathogenesis of emphysema. *Chest*. 1983;83(4):679–85.

80. Goldstein W, Döring G. Lysosomal enzymes from polymorphonuclear leukocytes and proteinase inhibitors in patients with cystic fibrosis. *Am. Rev. Respir. Dis.* 1986;134(1):49–56.

81. Suter S, Schaad UB, Morgenthaler JJ, Chevallier I, Schnebli HP. Fibronectin-cleaving activity in bronchial secretions of patients with cystic fibrosis. *J. Infect. Dis.* 1988;158(1):89–100.

82. Nakamura H, Yoshimura K, McElvaney NG, Crystal RG. Neutrophil elastase in respiratory epithelial lining fluid of individuals with cystic fibrosis induces interleukin-8 gene expression in a human bronchial epithelial cell line. *J. Clin. Invest.* 1992;89(5):1478–1484.

83. Khan TZ, Wagener JS, Bost T, Martínez J, Accurso FJ, Riches DW. Early pulmonary inflammation in infants with cystic fibrosis. *Am. J. Respir. Crit. Care Med.* 1995;151(4):1075–82.

84. Tosi MF, Zakem H, Berger M. Neutrophil elastase cleaves C3bi on opsonized pseudomonas as well as CR1 on neutrophils to create a functionally important opsonin receptor mismatch. *J. Clin. Invest.* 1990;86(1):300–308. doi:10.1172/JCI114699.

85. Voynow JA, Fischer BM, Zheng S. Proteases and cystic fibrosis. *Int. J. Biochem. Cell Biol.* 2008;40(6-7):1238–1245. doi:10.1016/j.biocel.2008.03.003.

86. Ballinger MN, Paine R, Serezani CHC, et al. Role of granulocyte macrophage colony-stimulating factor during gram-negative lung infection with Pseudomonas aeruginosa. *Am. J. Respir. Cell Mol. Biol.* 2006;34(6):766–774. doi:10.1165/rcmb.2005-0246OC.

87. Saha S, Doe C, Mistry V, et al. Granulocyte-macrophage colony-stimulating factor expression in induced sputum and bronchial mucosa in asthma and COPD. *Thorax*. 2009;64(8):671–676. doi:10.1136/thx.2008.108290.

88. Saba S, Soong G, Greenberg S, Prince A. Bacterial Stimulation of Epithelial G-CSF and GM-CSF Expression Promotes PMN Survival in CF Airways. *Am J Respir Cell Mol Biol*. 2002;27(5):561–567. doi:10.1165/rcmb.2002-0019OC.

89. Cox DR, Donnelly CA. *Principles of Applied Statistics*. 1st ed. Cambridge University Press; 2011.

90. Liou TG, Adler FR, Keogh RH, et al. Sputum biomarkers and the prediction of clinical outcomes in patients with cystic fibrosis. *Plos One*. 2012;7(8):e42748. doi:10.1371/journal.pone.0042748.

91. Davé SH, Tilstra JS, Matsuoka K, et al. Ethyl pyruvate decreases HMGB1 release and

ameliorates murine colitis. *J. Leukoc. Biol.* 2009;86(3):633 –643. doi:10.1189/jlb.1008662.

92. Korkmaz B, Attucci S, Epinette C, et al. Measurement of neutrophil elastase, proteinase 3, and cathepsin G activities using intramolecularly quenched fluorogenic substrates. *Methods Mol. Biol. Clifton Nj*. 2012;844:125–138. doi:10.1007/978-1-61779-527-5_9.

93. Kao RC, Wehner NG, Skubitz KM, Gray BH, Hoidal JR. Proteinase 3. A distinct human polymorphonuclear leukocyte proteinase that produces emphysema in hamsters. *J. Clin. Invest.* 1988;82(6):1963–73. doi:10.1172/JCI113816.

94. Lung function testing: selection of reference values and interpretative strategies. American Thoracic Society. *Am. Rev. Respir. Dis.* 1991;144(5):1202–18.

95. Harris PA, Taylor R, Thielke R, Payne J, Gonzalez N, Conde JG. Research electronic data capture (REDCap)--a metadata-driven methodology and workflow process for providing translational research informatics support. *J. Biomed. Inform.* 2009;42(2):377–381. doi:10.1016/j.jbi.2008.08.010.

96. Cox DR. Regression models and life-tables. *J. R. Stat. Soc. Ser. B Methodol.* 1972;34(2):187–220. doi:10.2307/2985181.

97. Cox DR, Oakes D. *Analysis of survival data*. CRC Press; 1984.

98. Sing T, Sander O, Beerenwinkel N, Lengauer T. ROCR: visualizing classifier performance in R. *Bioinformatics*. 2005;21(20):3940–3941. doi:10.1093/bioinformatics/bti623.

99. Davison AC, Hinkley DV. *Bootstrap Methods and Their Application*. 1st ed. Cambridge University Press; 1997.

100. Liou TG, Elkin EP, Pasta DJ, et al. Year-to-year changes in lung function in individuals with cystic fibrosis. *J. Cyst. Fibros. Off. J. Eur. Cyst. Fibros. Soc.* 2010;9(4):250–256. doi:10.1016/j.jcf.2010.04.002.
