# Supplementary material for: Prospective multicenter randomized patient recruitment and sample collection to enable future measurements of sputum biomarkers of inflammation in an observational study of cystic fibrosis
Source: BMC Med Res Methodol. 2019 Apr 26;19:88. doi: 10.1186/s12874-019-0705-0 (PMC6485181; doi:10.1186/s12874-019-0705-0)
Supplement: Supplementary file 7 — Sputum Processing Instructions. Provides the laboratory flow sheet for sample collection and illustrated instructions. (PDF 706 kb) [file 12874_2019_705_MOESM6_ESM.pdf]

SPUTUM PROCESSING INSTRUCTIONS

Protocol: Multicenter validation of predictive sputum biomarkers in CF

Site name: ______________________________________

Study ID number (from REDCap): __________________

Date sample collected: _____________ (DD/MMM/YYYY)

Time sample collected: _____:_____AM/PM

Specimen type: **Expectorated** ______ **Induced** ______

An adult Sample 1 must be expectorated.

DATA

Processing start time (HH:MM) _____:_____AM/PM

Weight of sample: ___ ___.___ ___ grams

Amount of HBSS from weight: ___ ___.___ ___ mL

Number of white capped cryovials: __________

Number of yellow capped cryovials: __________

Number of green capped cryovials: __________

Number of pink capped cryovials: __________

Signature: ________________________________

Date: ___________ (DD/MMM/YYYY)

COLLECTION

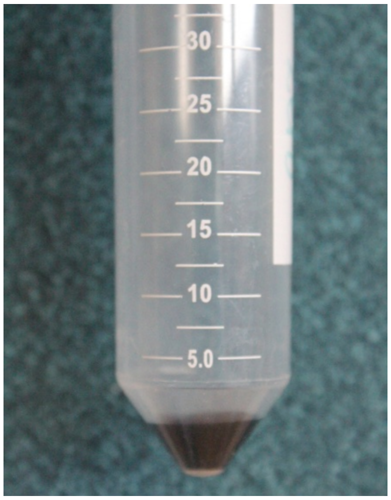

Picture 1: Bottom of this 50 mL tube has exactly 1 mL of dark blue fluid. Remember to correct for bubbles in sputum.

Collect sample by expectoration into 50 mL conical tube, alternate between two tubes to simultaneously collect clinical care and research samples.

Stop the collection of the clinical sample when there is enough for clinical care and continue just collecting research sample. Research samples need to be at least **1 mL** (see picture 1 to right) but encourage participant to try for **twenty minutes**—larger samples are preferred.

Sputum induction may be used as an alternative only if necessary, expectoration is preferred.

- *Do not do sputum induction unless it is usual clinical practice and is clinically indicated;* ***there is no study reimbursement for sputum induction****.*
- *Remember that the first adult sample* ***must*** *be expectorated.*

Put sample on ice immediately after collection and process within **4 hours** of sample collection.

- *Please do not process if the time from collection to processing exceeds 4 hours.*

Note: Before processing, remove a single use 15 mL aliquot of protease inhibitor cocktail from the freezer to thaw at room temperature. Once thawed, place on ice.

PROCESSING

1. Place an empty 50 mL tube on the scale and tare/zero the balance
2. Replace the empty tube with the sputum sample tube and record the weight of the sample
3. Dilute the sample 1:1 with HBSS using the sample weight to determine the mL of HBSS to add (1 gram of sputum = 1 mL of HBSS), record the mL of HBSS added
4. Vortex the sample at the highest speed for 1 minute (Picture 2)

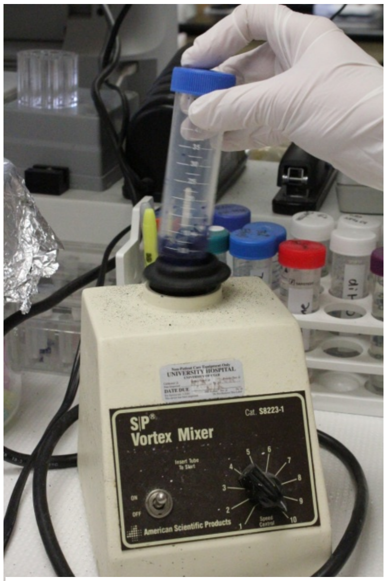

Picture 2

Picture 2 on left. Stop action photo! Sputum sample is to be vortex mixed for 1 minute.

Picture 3 on right. Pipetting 0.25 mL of blue fluid.

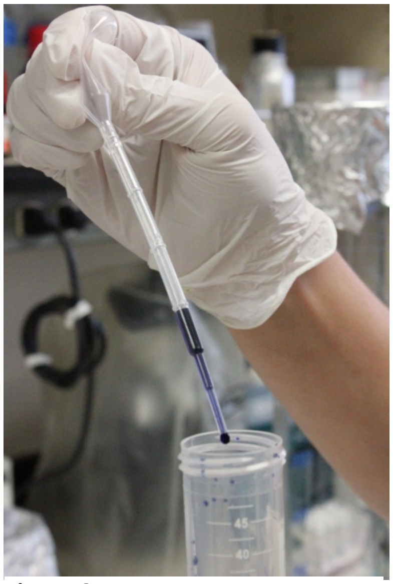

Picture 3

**ALIQUOT FOR CELL COUNTS**
5. Use a sterile pipette to remove 0.25 mL (Picture 3) of diluted sample from the conical tube and put into a 1.8 mL tube of any color which contains 0.25 mL Streck Solution. Mix gently with the sterile pipette. Send the 1.8 mL tube by FedEx within 24 hours.
**CENTRIFUGE**
6. Centrifuge the remaining sample in a 50 mL conical tube for 20 minutes at 2800 G at 4° Celsius (if possible, room temperature if not). *We will assist you with your calculation of G force if needed.*<br>After centrifugation, the sample should have two or three layers. There should be a pellet layer at the bottom, a fluid supernatant layer above the pellet, and *there may be a small lipid layer* above the supernatant (not all samples will have a lipid layer)

Centrifuged Sample

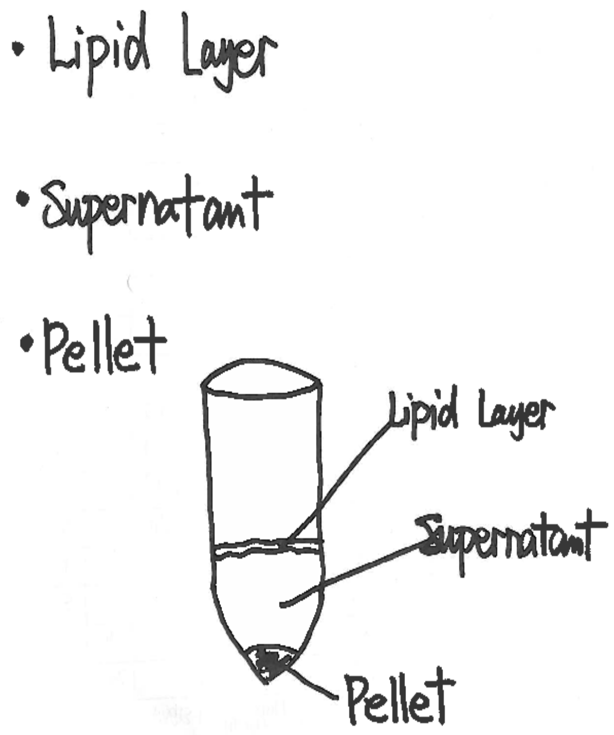

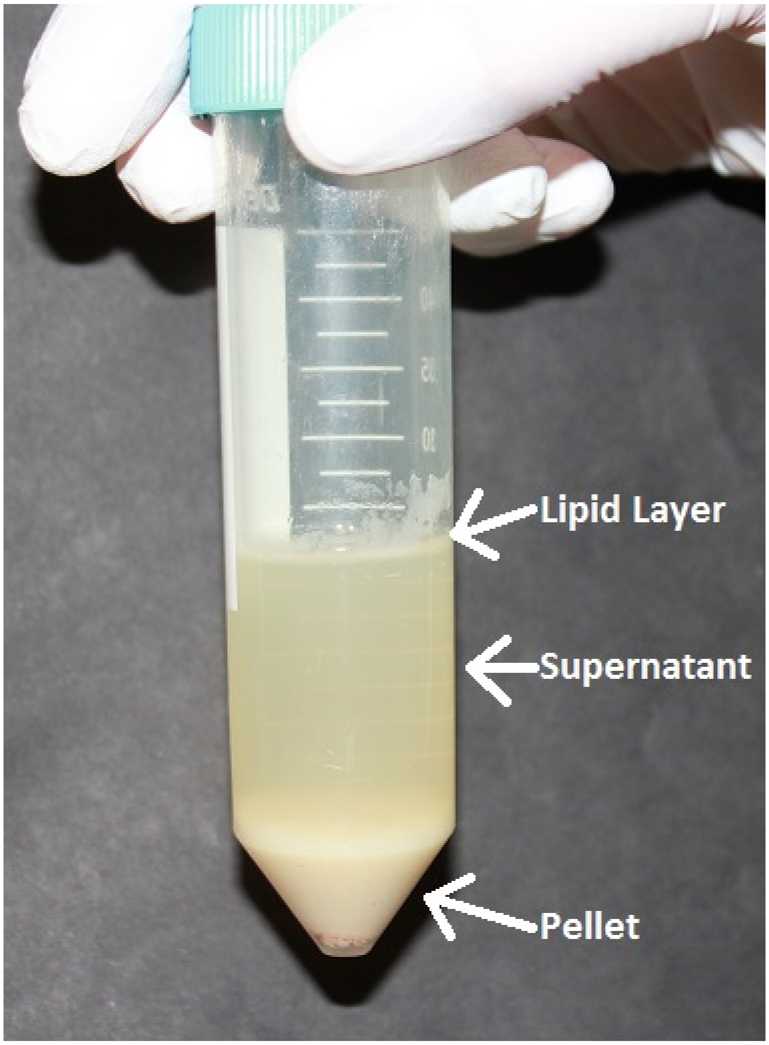

TRANSFER AND LABEL

7. Use a sterile pipette to transfer the **lipid layer** (if there is one) to clean 1.8 mL cryovials (picture 4, **white** capped lid). Label with study ID, date, "L" for lipid, and the sample number.

   Example label: *9999, 7/4/13, L1*
   Meaning: *Patient 9999, collected July 4th 2013; lipid layer from sample 1*

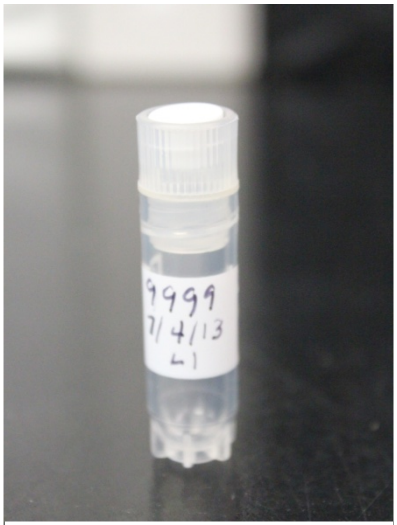

Picture 4

8. Use a sterile pipette to transfer the supernatant to a new sterile 15 mL conical tube labeled **SA**. *Take care to not contaminate the supernatant with pellet, it is better to leave a small amount of supernatant with the pellet than to contaminate the supernatant.* Save the tube containing the pellet but set aside to finish processing the supernatant.

9. Estimate the amount of supernatant using the graduation lines on the side of the conical tube and divide into two equal portions using another 15 mL conical tube. Label the second tube **SB**.

10. For **SA** add an equal volume of **HBSS**, vortex 10 seconds to mix, then pipette 1-1.5 mL aliquots into as many clean and sterile 1.8 mL cryovials as needed (picture 5, **yellow** capped lid). Write the study ID, date, SA, the sample number and the aliquot tube number on the side of the cryovials. SA will be used for neutrophil elastase activity measurements.

    Example: 9999, 7/4/2013, SA1, T5
    Meaning: patient 9999 collected 7/4/2013, Supernatant A fraction, Sample 1, Aliquot Tube 5

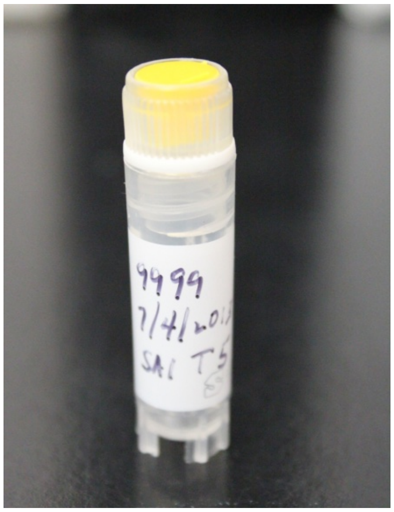

Picture 5

11. For **SB** add an equal volume of **protease inhibitor cocktail**, vortex 10 seconds to mix, then pipette 1-1.5 mL aliquots into as many clean and sterile 1.8 mL cryovials as needed (picture 6, **green** capped lid). Write study ID, date, SB, the sample number and the aliquot tube number on the side of the cryovials. SB will be used for HMGB-1 and GM-CSF measurements. **Please keep one SB vial separate from your batch shipments in case of shipment errors.**

    Example: 9999, 7/4/2013, SB1, T5
    Meaning: patient 9999 collected 7/4/2013, Supernatant B fraction, Sample 1, Aliquot Tube 5

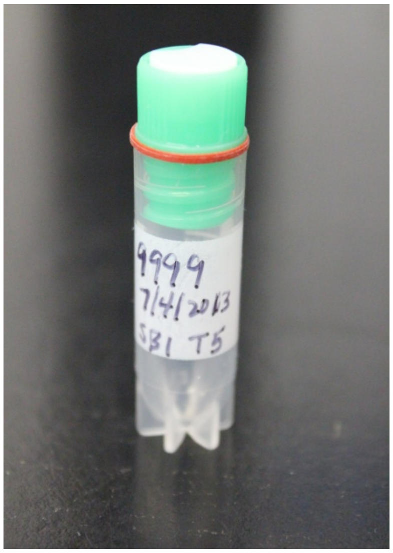

Picture 6

Note: The protease inhibitor cocktail cannot be re-frozen. Discard any unused cocktail after daily sample processing regardless of the amount left over.

12. Return to the tube containing the **pellet**. Use a sterile pipette and transfer 1-1.5 aliquots into clean and sterile 1.8 mL cryovials (picture 7, **pink** capped lid). Label with study ID, date, “P” for pellet, the sample number and aliquot tube numbers as needed. There will often be only 1 tube needed.

    Example: 9999, 7/4/2013, P1, T1
    Meaning: patient 9999 collected 7/4/2013, Pellet, Sample 1, Aliquot Tube 1

13. **Make sure to ship the cell count aliquot at room temperature by FedEx the same day as processing.**

14. **Keep one SB vial separate from the rest of your samples that will be batched shipped in case of shipping errors.** Store in -80ºC freezer with a copy of page 1 of these instructions.

15. Place all other samples in a biohazard bag with a copy of page 1 of these instructions. Store in -80ºC freezer to be batched shipped at later date.

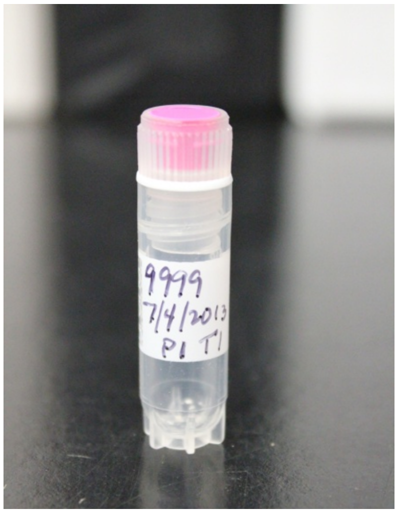

Picture 7
